# Aggregation-prone Tau impairs mitochondrial import, which affects organelle morphology and neuronal complexity

**DOI:** 10.1242/jcs.260993

**Published:** 2023-07-07

**Authors:** Hope I. Needs, Kevin A. Wilkinson, Jeremy M. Henley, Ian Collinson

**Affiliations:** School of Biochemistry, University of Bristol, Bristol BS8 1TD, UK

**Keywords:** Tau, Mitochondria, Mitochondrial import, Neurodegeneration, Neuronal complexity, Mitochondrial morphology

## Abstract

Mitochondrial protein import is essential for organellar biogenesis, and thereby for the sufficient supply of cytosolic ATP – which is particularly important for cells with high energy demands like neurons. This study explores the prospect of import machinery perturbation as a cause of neurodegeneration instigated by the accumulation of aggregating proteins linked to disease. We found that the aggregation-prone Tau variant (Tau^P301L^) reduces the levels of components of the import machinery of the outer (TOM20, encoded by *TOMM20*) and inner membrane (TIM23, encoded by *TIMM23*) while associating with TOM40 (*TOMM40*). Intriguingly, this interaction affects mitochondrial morphology, but not protein import or respiratory function; raising the prospect of an intrinsic rescue mechanism. Indeed, Tau^P301L^ induced the formation of tunnelling nanotubes (TNTs), potentially for the recruitment of healthy mitochondria from neighbouring cells and/or the disposal of mitochondria incapacitated by aggregated Tau. Consistent with this, inhibition of TNT formation (and rescue) reveals Tau-induced import impairment. In primary neuronal cultures, Tau^P301L^ induced morphological changes characteristic of neurodegeneration. Interestingly, these effects were mirrored in cells where the import sites were blocked artificially. Our results reveal a link between aggregation-prone Tau and defective mitochondrial import relevant to disease.

## Teaser

**Summary:** An aggregation-prone variant of Tau affects mitochondrial structure, correlates with reduced neuronal complexity, and invokes a rescue mechanism involving intercellular mitochondrial transport.

## INTRODUCTION

Mitochondrial protein import is a highly conserved process whereby most resident proteins are transported from where they are synthesised in the cytosol. It occurs through various pathways ensuring delivery to the desired location within the mitochondrion. The translocase of the outer membrane (TOM) complex acts as the major gateway for entry into the inter-membrane space (IMS). It consists of assembly and stability subunits (TOM5, TOM6 and TOM7; encoded by *TOMM5*, *TOMM6* and *TOMM7*, respectively), receptor subunits [TOM20, TOM22 and TOM70 (*TOMM20*, *TOMM22* and *TOMM70*)] and a central pore-forming subunit (TOM40; *TOMM40*) (Chacinska et al., 2009; Tucker and Park, 2019). Proteins traversing through TOM40 are then dispersed via distinct pathways, depending on their structure and target destination (Chacinska et al., 2009). The presequence pathway is used by precursor proteins with a cleavable N-terminal mitochondrial targeting sequence (MTS). These proteins are either laterally inserted into, or transported across, the inner mitochondrial membrane (IMM); respectively, through the translocase of the inner membrane sort (TIM23^SORT^) or motor (TIM23^MOTOR^) complexes. Cleavage of the MTS then enables the release and folding of the mature protein (Chacinska et al., 2009). These pathways (recently reviewed in Bar-Ziv et al., 2020; Harbauer et al., 2014; Needs et al., 2021) need to be tightly regulated, and subject to quality control, to maintain mitochondrial structure and function – which is required for the supply of energy (ATP), cellular homeostasis, and overall health (Sokol et al., 2014).

Mitochondrial dysfunction is a hallmark of neurodegeneration and is being increasingly linked to a failure of the protein import machinery (Lin and Beal, 2006; Schon and Przedborski, 2011). For instance, accumulated amyloid precursor protein (APP) within mitochondria from individuals with Alzheimer's disease interacts with the protein channel components of both outer and inner membranes: TOM40 and TIM23 (*TOMM40* and *TIMM23*) (Devi et al., 2006). Similarly, mitochondria isolated from the brains of people affected by Huntington's disease are decorated with the aggregation-prone variant of the Huntingtin protein (Htt). Again, Htt associates with the TIM23 complex and was shown to inhibit import as a result (Yano et al., 2014).

Neurofibrillary tangles (NFTs) comprise insoluble aggregations made up primarily of hyperphosphorylated Tau protein. NFTs are characteristic of Alzheimer's disease, as well as of all other primary and secondary tauopathies (Tapia-Rojas et al., 2019). Tau is highly abundant in neurons and is mostly localised in axons, where it binds to microtubules, promoting their assembly and stability (Binder et al., 1985; Black et al., 1996; Witman et al., 1976). Upon binding to microtubules, Tau modulates the activity of the motor proteins dynein and kinesin, which in turn regulates the axonal transport of cargo including mitochondria (Chaudhary et al., 2018; Dixit et al., 2008; Ebneth et al., 1998; Mandelkow et al., 2004; Stamer et al., 2002). Tau binding to microtubules is a highly dynamic process and is dependent on different Tau isoforms, mutations and post-translational modifications. Owing to the vital role of Tau in axonal migration, its aberrant behaviour can bring about neurological pathogenesis (Avila et al., 2004; Barbier et al., 2019; Guo et al., 2017; Szabo et al., 2020). The Tau^P301L^ variant is commonly found in individuals with tauopathies and has been well-characterised in disease models (Goedert and Jakes, 2005; Poorkaj et al., 2001). Critically, Tau^P301L^ has an increased propensity to form NFTs (Murakami et al., 2006).

Mitochondrial function is impaired by abnormal pathological Tau, but the mechanism is not fully understood. Tau accumulation into NFTs can elicit several specific mitochondrial effects. These include increased retrograde transport of mitochondria (i.e. towards the soma); reduced complex I activity, ATP levels and membrane potential; enhanced oxidative stress; and defective mitophagy (Hu et al., 2016; Lasagna-Reeves et al., 2011; Li et al., 2016; Stamer et al., 2002). Furthermore, various studies have shown that aggregation-prone Tau accumulates within the IMS and outer mitochondrial membrane (OMM) (Cieri et al., 2018; Hu et al., 2016). This build-up and consequent alterations in membrane potential suggest that there might also be protein import defects (membrane potential is required for the translocation of proteins across the IMM). However, the effects of pathological Tau on the import process have yet to be examined.

Here, we show that the disease-linked variant Tau^P301L^ interacts with the channel-forming subunit of the TOM complex (TOM40). The association perturbs the import function and corresponds to changes in mitochondrial morphology and reduced neuronal complexity. Interestingly, the changes in mitochondrial morphology and neuronal structure match the changes seen when mitochondrial import sites are artificially blocked (see also Needs et al., 2022 preprint). These data suggest that perturbation of mitochondrial protein import, specifically by blockage of the translocation machinery by variants of Tau, or other aggregation-prone proteins, might be an important factor in the onset of neurodegenerative diseases.

## RESULTS

### The aggregation-prone variant Tau^P301L^ associates with the mitochondrial import machinery

**Fig. 1.**
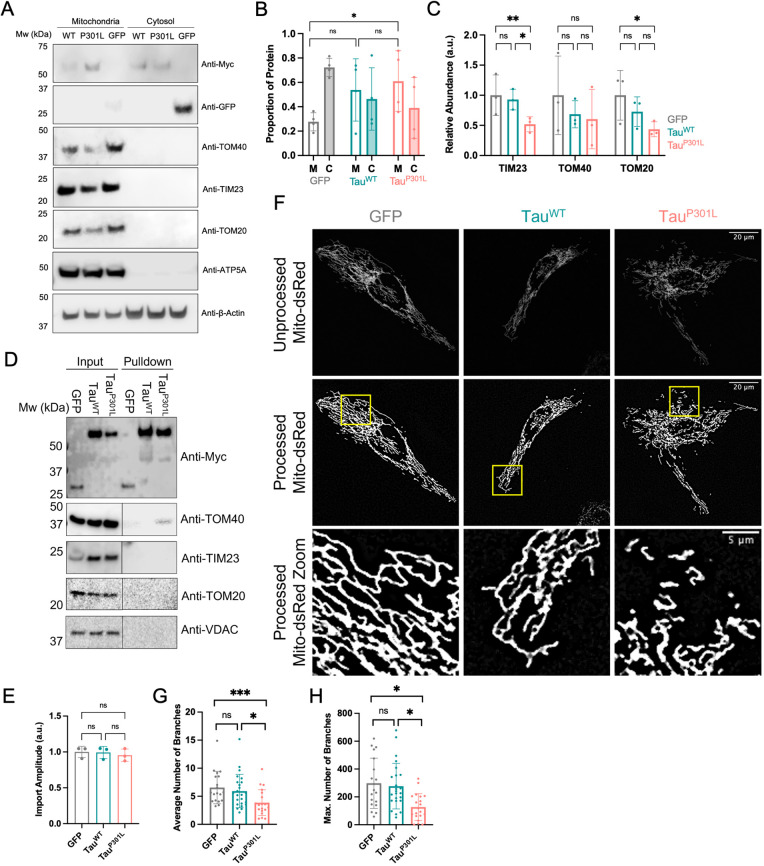
**Tau^P301L^ associates with the mitochondrial translocation machinery and alters mitochondrial morphology.** (A) Representative western blots showing relative protein abundance in mitochondrial and cytosolic fractions of HeLaGAL cells producing GFP, Myc–Tau^WT^ or Myc–Tau^P301L^ (following lentiviral infection). Localisation of GFP, Tau^WT^ and Tau^P301L^ was analysed by probing for GFP (Sigma; G1544; 1:1000) and Myc (CST; 2276; 1:5000). Translocase subunit abundance was analysed by probing for TOM40 (Thermo Fisher Scientific; PA5-57575; 1:1000), TIM23 (Invitrogen; PA5-71877; 1:1000) and TOM20 (Santa Cruz Biotechnology; sc-17764; 1:500). ATP5A (Abcam; 15H4C4; 1:1000) and β-actin (Sigma; A2228; 1:10000) were used as loading controls for the mitochondria and cytosol, respectively. *N*=4 (GFP/Myc) and *N*=3 (translocases) biological replicates. (B) Quantification of the proportion of GFP, Tau^WT^ or Tau^P301L^ in mitochondrial (labelled M) and cytosolic (labelled C) fractions of HeLaGAL cells. Band intensity values were first normalised to their respective mitochondrial/cytosolic loading controls, summed to obtain the ‘total’ abundance, and the mitochondrial/cytosolic fraction was determined as a proportion of the total (mitochondrial/total or cytosolic/total). Error bars show s.d. One-way ANOVA and Tukey's post hoc test were used to determine significance. *P*-values (left to right, bottom to top): >0.9999, 0.5648, 0.0135. (C) Quantification of the relative abundance of translocase subunits (TIM23, TOM40 and TOM20) in HeLaGAL cells over-producing GFP, Tau^WT^ or Tau^P301L^. Normalised to loading controls. Error bars show s.d. One-way ANOVA and Tukey's post hoc test were used to determine significance. *P*-values (left to right, bottom to top): TIM23: 0.0538, 0.0273, 0.0014; TOM40: 0.4785, 0.9474, 0.3362; TOM20: 0.2166, 0.3810, 0.0356. (D) Representative western blot showing TOM40 association with Tau^P301L^, but not with Myc-tagged GFP or Tau^WT^, in the mitochondrial fraction of HeLaGAL cells. Mitochondria were isolated from HeLaGAL cells over-producing Myc–GFP, Myc–Tau^WT^ or Myc–Tau^P301L^ and proteins were solubilised gently with GDN (input) before immunoprecipitation using Myc-trap beads (Chromotek). Eluted proteins (pulldown) were analysed by western blotting and probed against Myc (to validate IP) and TOM40, TIM23, and TOM20, to observe possible association of Tau variants with import machinery. VDAC (Invitrogen; PA1-954A; 1:1000) was used as a mitochondrial loading control. The input represents 25% of the lysate used in the pulldown. The input and pulldown samples were run on the same gel, but a higher exposure was used for the pulldown for TOM40, TOM20, TIM23 and VDAC blots, to detect low levels of binding. This is highlighted by a line in the respective panels, and both low and high exposures are shown in Fig. S4. *N*=3 biological replicates. Quantification of the TOM40–Tau interaction is shown in Fig. S1B. (E) Maximum import amplitude from MitoLuc import assay on HeLaGAL cells over-producing GFP, Tau^WT^ or Tau^P301L^. Bars represent the relative maximum mitochondrial import of the precursor protein *Su9-EGFP-pep86* [normalised to eqFP670 expression, maximum amplitude for run, and control (GFP)]. *N*=3 biological repeats, each with *n*=3 technical replicates. Error bars display s.d. One-way ANOVA with Tukey's post hoc test was used to determine significance. Import traces are shown in Fig. S1C. *P*-values (left to right, bottom to top): 0.9997, 0.9568, 0.9492. (F) Representative confocal images showing mitochondrial morphology in HeLaGAL cells expressing mito-dsRed and GFP (left, grey), Tau^WT^ (middle, teal) or Tau^P301L^ (right, pink). The top panel shows mito-dsRed (mitochondria) before processing. The middle panel shows mitochondria after processing, and the bottom panel shows a zoom of an area of mitochondria (highlighted in the middle panel yellow box) to give a clear view of mitochondrial morphology. *N*=5 biological repeats. (G) Quantification of the average number of mitochondrial branches in a network. Nested one-way ANOVA and Tukey's post hoc test were used to determine significance. Statistics were carried out on *N*=5 biological replicates. Each point on the graph represents an individual cell to display the spread of the data (20, 24, and 23 cells were counted for each condition, respectively). Error bars show s.d. *P*-values (left to right, bottom to top): 0.2624, 0.0345, 0.0004. (H) Quantification of the maximum number of mitochondrial branches in a network. Statistical analysis is exactly as described in G*. P*-values (left to right, bottom to top): 0.9886, 0.0395, 0.0321. ns, *P*>0.05, *P≤0.05, **P≤0.01, ***P≤0.001. a.u., arbitrary units.

The association of different Tau variants [native (Tau^WT^) and disease-linked (Tau^P301L^)] with mitochondria was investigated in HeLa cells conditioned with galactose (HeLaGAL). Galactose impairs glycolytic metabolism and drives cells to be more dependent on mitochondria for their energy supplies (Aguer et al., 2011). We produced Myc–Tau^WT^, Myc–Tau^P301L^ or a GFP control within these cells by lentiviral infection and carried out western blot analysis on the fractionated mitochondria and cytosol (Fig. 1A). The results show that, in cells containing Myc–Tau^P301L^, a higher proportion of the over-produced protein localised to mitochondria compared to in the GFP control (Fig. 1A, quantified in B). Together with confocal imaging data (Fig. S1A), it is clear that a small, but potentially critical, pool of Tau (both wild-type and disease-prone variants) localises to the mitochondria.

To test whether the increased mitochondrial localisation of Tau^P301L^ correlated with altered import pathway components, we expanded this analysis to include a selection of subunits of the import machinery (Fig. 1A, quantified in 1C). Overall, mitochondria from cells over-producing Tau^P301L^ had lower levels of mitochondrial TOM20 (a receptor subunit of the TOM complex of the OMM) and TIM23 (a channel-forming subunit of the TIM23 complex in the IMM) compared to control cells. There was no change in the abundance of TOM40 (channel-forming subunit of TOM complex of the OMM). Together, these data are indicative of potential mitochondrial import defects induced by Tau^P301L^.

Next, we investigated the interaction between Tau^P301L^ and the translocation machinery using a Myc-trap on mitochondrial extracts from HeLaGAL cells producing recombinant Myc–GFP, Myc–Tau^WT^ or Myc–Tau^P301L^ (cells transduced by lentiviral infection). Western blots of the Myc pulldowns showed that Tau^P301L^, but not its native counterpart or the GFP control, enabled the recovery of detectable quantities of TOM40 (but not TIM23, TOM20 or VDAC; Fig. 1D, quantified in Fig. S1B). This is consistent with previous reports that showed Tau^P301L^ accumulation in the OMM and IMS (Cieri et al., 2018; Hu et al., 2016) and suggests that Tau^P301L^ might associate with the translocation channel in a similar way to an artificially trapped precursor protein (Chacinska et al., 2003; Ford et al., 2022; Needs et al., 2022 preprint). Surprisingly, however, despite the association of Tau^P301L^ with the import machinery, in-cell MitoLuc import assays (Needs et al., 2023) showed that the import kinetics of a precursor reporter were not significantly affected by transient expression of Tau^P301L^ compared to Tau^WT^ or the positive control, GFP (Fig. 1E; Fig. S1C).

Analysis of mitochondrial branching showed that cells transfected with the construct encoding Tau^P301L^ had significantly fewer branches for each mitochondrial network when compared to mitochondria from cells producing Tau^WT^ or GFP (3.9 compared to 5.9 and 6.6, respectively; Fig. 1F,G). The maximum number of branches for each network was also reduced to 127.3 for Tau^P301L^, compared to 276.9 for Tau^WT^ and 298.3 for GFP (Fig. 1H). Mitochondrial stress tests measuring oxygen consumption and membrane potential (TMRM fluorescence) showed that, despite Tau^P301L^-induced alterations in mitochondrial morphology, the respiratory capacity was unaffected by the presence of either the native (WT) or aggregation-prone (P301L) Tau variants (Fig. S1D–F).

### Over-production of the variant Tau^P301L^ induces TNT formation

In a parallel study, we have explored the effects of artificially blocking the import machinery (Needs et al., 2022 preprint). This can be achieved with the small-molecule inhibitor MB20 (Cheung, 2017) or with a precursor fused to dihydrofolate reductase (DHFR), which, when bound to methotrexate (MTX), becomes trapped in the import channel (Chacinska et al., 2003; Ford et al., 2022). Although these treatments inhibit import in isolated mitochondria (Cheung, 2017; Ford et al., 2022), we unexpectedly found that they fail to do so within whole cells (Needs et al., 2022 preprint).

We wondered whether the apparent lack of effect on the whole mitochondrial population could be explained by a mitochondrial replacement system. We found that, at the whole-cell level, defective mitochondrial function is rescued by the recruitment of healthy mitochondria from surrounding cells, and the disposal of compromised mitochondria, by way of TNTs (Needs et al., 2022 preprint). We, therefore, tested whether the association of the Tau variant with the import apparatus induced a similar response.

**Fig. 2.**
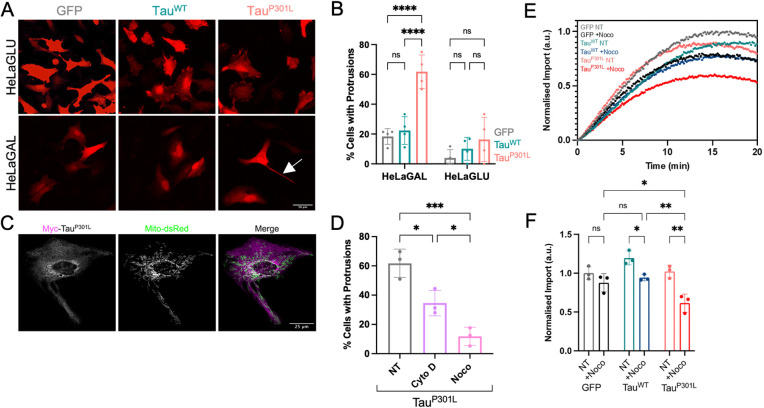
**HeLaGAL cells over-producing Tau^P301L^ form TNTs that contain mitochondria.** (A) Representative confocal images demonstrating the cell morphology of HeLaGLU or HeLaGAL cells subjected to over-production of mCherry (red) as well as GFP, Tau^WT^, or Tau^P301L^. The white arrow in the bottom right panel indicates a TNT. *N*=4 biological replicates. (B) Quantification of the proportion of cells with protrusions from representative images shown in A. Error bars show s.d. Two-way ANOVA and Šidák's multiple comparison tests were used to determine significance*. N*=4 biological replicates, 20 cells were counted per replicate. *P*-values (left to right, bottom to top): HeLaGAL, 0.9145, <0.0001, <0.0001; HeLaGLU, 0.7546, 0.7435, 0.2265. (C) Representative confocal images showing mitochondrial localisation (mito-dsRed; green) in cells over-producing Myc–Tau^P301L^ [magenta; anti-Myc (Cell Signalling Technologies; 2276; 1:1000)]. *N*=4 biological replicates. (D) Quantification of the percentage of HeLaGAL cells with protrusions following Tau^P301L^ over-production in the presence of TNT inhibitors. HeLaGAL cells were subjected to over-production of Tau^P301L^–mScarlet and either untreated (NT; DMSO only) or treated with 50 nM cytochalasin D or 100 nM nocodazole for 48 h. *N*=3 biological replicates, 20 cells counted per replicate. Error bars show s.d. One-way ANOVA and Tukey's post hoc test were used to determine significance. Representative images are shown in Fig. S2B. *P*-values (left to right, bottom to top): 0.0169, 0.0357, 0.0008. (E) MitoLuc import traces showing the impact of the TNT inhibitor nocodazole on the import of a precursor protein in HeLaGAL cells over-producing Tau variants. Cox8a-11S-producing cells were transfected with GFP, Tau^WT^ or Tau^P301L^ in the presence (+Noco) or absence (NT) of 100 nM nocodazole for 48 h before monitoring the import of *Su9-EGFP-pep86* by MitoLuc import assays. *N*=3 biological replicates. Data are normalised to the maximum signal. (F) Quantification of import amplitude for import traces shown in Fig. 2E. Data are normalised to untreated GFP control. Error bars represent s.d. Two-way ANOVA with Tukey's post hoc test was used to determine significance. *P*-values (left to right, bottom to top): 0.5819, 0.0474, 0.0016, 0.9457, 0.0092, 0.0401. ns, *P*>0.05, *P≤0.05, **P≤0.01, ***P≤0.001; ****P≤0.0001. a.u., arbitrary units.

HeLa cells co-transfected with mCherry and Myc–GFP, Myc–Tau^WT^ or Myc-Tau^P301L^ were analysed by confocal microscopy. In mitochondrial-dependent HeLa cells conditioned with galactose (HeLaGAL), production of Myc–Tau^P301L^ induced TNTs – 62% of all cells exhibited TNTs, compared to 22% of cells producing native Tau and 18% of those expressing GFP alone (Fig. 2A,B). Staining with wheat germ agglutin (WGA) highlighted that the protrusions formed in response to HeLaGAL cell transfection with Tau^P301L^ were long, thin and membranous protrusions connecting neighbouring cells, consistent with the definition of a TNT (Fig. S2A). In contrast, glycolytic HeLaGLU cells (cultured in glucose-containing medium) were substantially less prone to TNT production. These observations could explain why the association of Tau^P301L^ with TOM40 has no impact on protein import activity; that is, by way of the induction of a TNT-dependent rescue mechanism for replenishment of defective mitochondria in those cells producing Tau^P301L^.

Consistent with this hypothesis, we observed mitochondria inside the TNTs (Fig. 2C), which we hypothesise are healthy mitochondria from neighbouring healthy cells, migrating to rescue Tau-affected cells with compromised mitochondria. TNTs might also enable the transfer of damaged mitochondria, with accumulated Tau aggregates, to a neighbouring cell for degradation.

It is notable that in these confocal microscopy data, there is sparse colocalisation apparent between Tau and the mitochondria. However, this is not unexpected given that Tau has major roles in microtubule stabilisation, and we only observed a very small proportion of Tau associating with mitochondria in our biochemical analyses. Note that the representative cell shown in Fig. 2C is the same cell shown in Fig. 1F.

TNTs induced by import impairment contain actin as well as microtubules, both of which are necessary for mitochondrial movement (Needs et al., 2022 preprint). Thus, drugs that target actin (cytochalasin D) and tubulin (nocodazole) polymerisation inhibit TNT formation (Bittins and Wang, 2017; Kumar et al., 2017; Luchetti et al., 2012). The TNTs induced here also contain actin and tubulin, and display a similar drug response (Fig. 2D; Fig. S2B). Therefore, perturbation of the import machinery by precursor trapping, small-molecule inhibition [shown previously (Needs et al., 2022 preprint)] and, as we show here, aggregation of Tau, seem to induce a common response involving TNTs. Importantly, when cells were treated with the TNT inhibitor nocodazole, mitochondrial import became sensitive to Tau^P301L^, and to a lesser extent, Tau^WT^ (Fig. 2E,F) owing to inactivation of rescue. These data highlight the role of mitochondrial transfer via TNTs in rescuing the import function of cells exposed to mitochondrial Tau accumulation.

### Both Tau^P301L^ over-production and precursor trapping reduce neuronal complexity

Next, we explored the effects of perturbing the mitochondrial import machinery in neurons. In particular, we compared the effects of precursor trapping against the production of Tau^P301L^. Precursor proteins are imported in an unfolded state, so the C-terminal fusion of DHFR, which resists unfolding when bound to the drug MTX, can be exploited to block the import machinery (Chacinska et al., 2003; Eilers and Schatz, 1986; Ford et al., 2022; Rassow et al., 1989). We used confocal microscopy to assess the morphology of DIV21 (21 days *in vitro*) primary hippocampal neurons following 7 days of precursor trapping or Tau variant over-production by transient transfection. DIV21 neurons equate to ‘mature’ neurons with spines and fully formed synapses (Grabrucker et al., 2009; LaBarbera et al., 2021), and therefore allow an analysis of synapses and neuronal complexity. Axons were identified by staining for ankyrin-G, a scaffolding protein important for the formation of the initial segment of the axon (Grubb and Burrone, 2010).

**Fig. 3.**
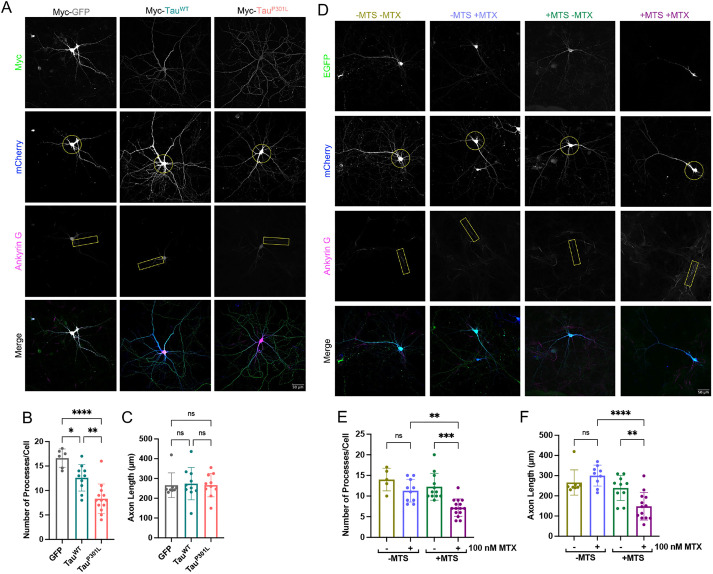
**Tau^P301L^ and precursor trapping reduce neuronal complexity.** (A) Representative confocal images showing the morphology of DIV21 primary hippocampal neurons over-producing mCherry (whole-cell cytosolic marker; blue; circle used to highlight the complexity in the second panel down, i.e. the number of processes that cross the circle is representative of the number of processes per cell) and Myc-tagged GFP, Tau^WT^ or Tau^P301L^ (Myc; green; Cell Signalling Technologies 2276; 1:1000). Axon initial segments were stained for ankyrin-G (magenta; highlighted by the box in the third panel down; NeuroMab 75-146; 1:400). *N*=4 biological replicates. (B) Quantification of the number of processes per cell for neurons over-producing GFP, Tau^WT^ or Tau^P301L^. Each data point represents an individual cell (cells were only analysed if the entire cell could be identified separately from surrounding cells). Error bars show s.d. Nested one-way ANOVA and Tukey's post hoc test were used to determine significance. *P*-values (left to right, bottom to top): 0.0219, 0.0025, <0.0001. (C) Quantification of the axon length for neurons over-producing GFP, Tau^WT^ or Tau^P301L^. Cells were only analysed if the axon could be clearly identified. Error bars show s.d. Nested one-way ANOVA and Tukey's post hoc test were used to determine significance. *P*-values (left to right, bottom to top): 0.9688, 0.9703, 0.9998. (D) Representative confocal images showing the morphology of DIV21 primary hippocampal neurons over-producing mCherry (whole-cell cytosolic marker; blue; circle used to highlight the complexity in the second panel down, i.e. the number of processes that cross the circle is representative of the number of processes per cell) as well as EGFP–DHFR (−MTS; green; top panel) or Su9-EGFP–DHFR (+MTS; green; top panel) in the presence or absence of 100 nM MTX. Axons were stained with ankyrin-G (magenta; highlighted by the box in the third panel down; NeuroMab 75-146; 1:400). *N*=5 biological replicates. (E) Quantification of the number of processes per cell in neurons over-producing EGFP–DHFR (−MTS) or Su9-EGFP–DHFR (+MTS) in the presence or absence of 100 nM MTX. Each data point represents an individual cell (cells were only analysed if the entire cell could be identified separately from surrounding cells). Error bars show s.d. Nested one-way ANOVA and Tukey's post hoc test were used to determine significance. *P*-values (left to right, bottom to top): 0.2769, 0.0001, 0.0034. (F) Quantification of axonal length in neurons over-producing EGFP–DHFR (−MTS) or Su9-EGFP–DHFR (+MTS) in the presence or absence of 100 nM MTX. Cells were only analysed if the axon could be clearly identified. Error bars show s.d. Nested one-way ANOVA and Tukey's post hoc test were used to determine significance. *P*-values (left to right, bottom to top): 0.6700, 0.0071, <0.0001. ns, *P*>0.05, *P≤0.05, **P≤0.01, ***P≤0.001; ****P≤0.0001.

Over-production of native Tau caused a significant loss of the number of processes per cell compared to what was seen upon expression of the GFP control, whereas expression of the aggregation-prone Tau^P301L^ caused a more pronounced reduction in the number of processes (Fig. 3A,B). Despite the reduction in neuronal complexity, neither Tau variant affected axonal length (Fig. 3C). To determine whether these effects could be attributed to perturbation of the import machinery by Tau, we also subjected primary neurons to mitochondrial precursor trapping. The inclusion of the precursor alone did not affect cell complexity, but when the import of this precursor was stalled by MTX addition, the number of processes was significantly reduced (Fig. 3D,E), mirroring the effect of Tau. In addition, and in contrast to the effects of Tau, stalling import significantly reduced axonal length (Fig. 3F; +MTS, +MTX), suggesting a more severe response. Importantly, the changes in length and complexity were not due to non-specific effects of MTX, as they are dependent on the MTS (Fig. 3E,F; −MTS, ±MTX). Neuronal viability was unaffected by precursor trapping (±MTS) or production of Tau variants, although there was a measurable effect of the MTX drug, which is inconsequential for this analysis (Fig. S3A,B).

These results indicate that a reduction in neuronal complexity and length is elicited by perturbation of the mitochondrial protein import machinery. Notwithstanding the differing response to axonal length, this morphological change can be induced either by artificial precursor stalling or Tau production – most severely with the aggregation-prone variant. Therefore, we assume this effect is mediated via a common mechanism, initiated by an interaction with TOM40.

### Precursor trapping and production of Tau^P301L^ reduce the number of synapses

The neuronal analysis was expanded to investigate the number of synapses. The major scaffold protein PSD95 (also known as DLG4 or SAP90) is involved in the organisation of excitatory postsynaptic signalling complexes in neurons (Broadhead et al., 2016), and is a widely used postsynaptic marker. These signalling complexes comprise glutamate receptors, ion channels, signalling enzymes and adhesion proteins vital for neurotransmission (Chen et al., 2011).

**Fig. 4.**
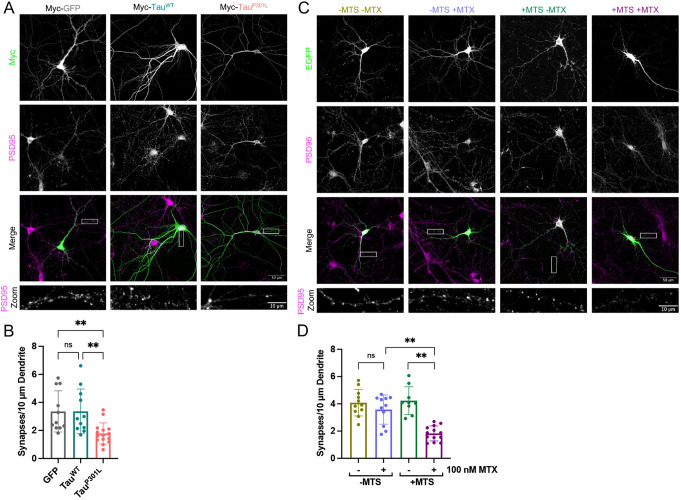
**Tau^P301L^ and precursor trapping reduce synapse abundance.** (A) Representative confocal images showing synaptic staining of DIV21 primary hippocampal neurons over-producing Myc-tagged GFP, Tau^WT^ or Tau^P301L^ (Myc; green; Cell Signaling Technologies 2276; 1:1000). PSD95 staining shows synapses (magenta; second panel from the top, also shown in the zoom; EMD Millipore AB1596; 1:400). *N*=5 biological replicates. (B) Quantification of the average number of synapses per 10 µm dendrite for GFP, Tau^WT^ and Tau^P301L^ over-producing neurons. Synapses were counted manually on anonymised data. Each data point represents an individual cell. Error bars show s.d. Nested one-way ANOVA and Tukey's post hoc test were used to determine significance. *P*-values (left to right, bottom to top): >0.9999, 0.0098, 0.0100. (C) Representative confocal microscopy images showing synaptic staining of DIV21 primary hippocampal neurons over-producing EGFP–DHFR (−MTS) or Su9-EGFP–DHFR (+MTS; EGFP shown in green, top panels) in the absence or presence of 100 nM MTX (±MTX). PSD95 staining shows synapses (magenta, second panel from the top, also shown in the zoom; EMD Millipore AB1596; 1:400). *N*=5 biological replicates. (D) Quantification of the average number of synapses per 10 µm dendrite for neurons over-producing EGFP–DHFR (−MTS) or Su9-EGFP–DHFR (+MTS) in the presence or absence of 100 nM MTX. Synapses were counted manually on anonymised data. Each data point represents an individual cell. Error bars show s.d. Nested one-way ANOVA and Tukey's post hoc test were used to determine significance. *P*-values (left to right, bottom to top): 0.8172, 0.0015, 0.0090. ns, *P*>0.05, **P≤0.01.

Imaging and PSD95 staining showed that transfected neurons producing Tau^P301L^ contained an average of 1.8 synapses per 10 µm dendrite, which is significantly lower than the number in cells expressing GFP or native Tau, both of which had about twice as many (Fig. 4A,B). Subsequent western blot analysis of whole-cell lysates from cortical cells over-producing GFP, or either of the Tau variants, transduced by lentiviral infection, determined that there was no change in the overall abundance of the synaptic markers PSD95, gephyrin or synaptophysin (Fig. S3C–F). This indicates that the noted reduction in synapse abundance is likely due to a redistribution of PSD95 rather than its overall quantity.

Perturbation of the import machinery by precursor trapping induced an almost identical reduction in the number of synapses per dendrite to the production of Tau^P301L^ (Fig. 4C,D). Again, the controls show that synaptic loss is correlated to the precursor trapping and not MTX treatment, given that in the absence of a presequence (−MTS) there was no significant change in the number of synapses in the presence of MTX (Fig. 4D, −MTS, ±MTX). Western blot analysis showed that the overall cellular abundance of all three synaptic marker proteins remained constant irrespective of precursor trapping (Fig. S3G–J), as noted above for the Tau variants.

These results demonstrate that over-production of aggregation-prone Tau^P301L^, which binds to and impairs the mitochondrial import machinery, produces defects in neuronal complexity and synapse number that are similar to those observed upon direct blockade of import.

## DISCUSSION

This study explores aspects of the mitochondrial and cellular responses to Tau, focusing particularly on the aggregation-prone Tau^P301L^ implicated in neurodegeneration. We established that the association of Tau^P301L^ with the mitochondrial protein import machinery correlates with disease-like phenotypic changes in mitochondria and neurons. Moreover, the effects of Tau mirror those elicited by a precursor protein artificially stalled within the import machinery; suggesting that Tau^P301L^ also exerts its effects through perturbation of the import machinery.

At the mitochondrial level, import perturbation by various means causes similarly profound morphological changes including decreased branching (Tau aggregation) and increased fission (precursor trapping) (Needs et al., 2022 preprint). Unexpectedly, however, neither Tau^P301L^ nor precursor trapping affected the overall mitochondrial import activity. We postulate that this can be explained by both Tau^P301L^ and precursor trapping inducing the formation of actin- and tubulin-containing tunnelling nanotubes (TNTs) (Needs et al., 2022 preprint).

It has been shown in several previous studies that TNTs can provide a conduit for the exchange of mitochondria between cells [as summarised in a recent review (Liu et al., 2021)]. Thus, we propose that healthy mitochondria are recruited to damaged cells via TNTs from nearby unaffected (healthy) cells. Damaged mitochondria are also likely concurrently disposed of through TNTs, as we recently showed following precursor trapping (Needs et al., 2022 preprint). This process could also provide a rescue mechanism that explains the observed preservation of mitochondrial import activity and respiratory function despite Tau^P301L^ accumulation at the TOM40 import channel. Indeed, this is consistent with our subsequent MitoLuc data showing that Tau (particularly Tau^P301L^) reduces import efficiency when TNT formation is restricted. This present study has focused on the effects of Tau variants on import via the presequence pathway into the matrix (TIM23^MOTOR^); its effect on the alternative pathways has yet to be formally assessed. Given that that blockage occurs at TOM40, it is likely that the other pathways to the IMS [mitochondrial IMS assembly (MIA)] and into the inner membrane (TIM23^SORT^ and TIM22) will also be affected.

The Tau variant-induced neuronal changes seen here recapitulate those observed after precursor trapping, strongly suggesting that they occur, at least in part, as a result of perturbation of the import machinery. In this context, we do not yet know whether the TNT-dependent rescue response, noted in mitochondria-dependent HeLa cells, occurs in neurons. However, it has been shown that neurons form TNTs, and these transport Tau (Tardivel et al., 2016). Perhaps the rescue mechanism described here incorporates, in addition to intercellular mitochondrial sorting, routes for the clearance of pathological proteins from critical cells like neurons.

The decrease in dendritic and axonal processes, and the reduced synapse number induced by perturbation of the import machinery are consistent with changes observed in neurodegeneration (Coleman and Perry, 2002; Kweon et al., 2017; Wishart et al., 2006). Therefore, an important future goal is to determine whether the TNT rescue mechanism is implemented in neurons exposed to import defects and, if so, to what extent it mitigates against more severe neuronal aberrations or cell death. Indeed, we conjecture that the absence of an impact of Tau^P301L^ or precursor trapping on cell viability could have been due to the rescue process. It is important to note that the association of Tau^P301L^ with the import machinery was shown in HeLa cells and not in neurons, and as such the implications for neurons concerning import dysfunction are solely correlative.

The variant Tau^P301L^ has been extensively characterised (Goedert and Jakes, 2005; Murakami et al., 2006; Poorkaj et al., 2001) and, in agreement with the results shown here, it has previously been shown to localise to the mitochondrial IMS and OMM (Cieri et al., 2018; Hu et al., 2016). However, its interaction with the translocation machinery had not previously been reported. Nonetheless, its association with mitochondrial translocases is reminiscent of other proteins implicated in neurodegeneration, such as APP, huntingtin and α-synuclein (Devi et al., 2006, 2008; Yano et al., 2014). Therefore, the neuronal effects of import perturbation shown here point to a common mechanism that could be an important underlying factor in multiple neurological diseases. Indeed, reports have linked failed import or aggregation of proteins within import sites with neurodegenerative diseases – both aggregation-prone APP and a disease-associated huntingtin protein variant become trapped in import sites and are directly correlated with mitochondrial dysfunction, a characteristic feature of neurodegeneration (Devi et al., 2006; Yano et al., 2014).

The exact mechanism linking import perturbation and neuronal irregularities, including the role of TNTs and rescue, remains unclear. Nevertheless, the evidence suggests that our artificial trapping substrate is indeed mimicking what is seen in disease. Therefore, it provides a useful tool to better understand import dysfunction as a potential contributory factor in the onset of neurodegenerative disease. In the future, it will be useful to expand this work with a more thorough analysis of the neuronal mitochondrial proteome, to gain a better understanding of the effects of Tau and its potential interacting partners. Certainly, it will be interesting to determine how the levels of the TOM and TIM complexes change in response to the mitochondrial association of Tau. Moreover, a deeper analysis of the potential TNT-mediated rescue mechanism should be pursued, particularly with regard to early and late-onset neurodegeneration, and perhaps how these pathways could be manipulated to delay the progression of disease.

## MATERIALS AND METHODS

### Reagents

All chemicals were of the highest grade of purity commercially available and purchased from Sigma, UK unless stated otherwise. Aqueous solutions were prepared in ultrapure water; for non-aqueous solutions, ethanol or DMSO was used as the solvent instead. Antibody catalogue numbers and suppliers are detailed in figure legends. Antibodies were all commercially available and used following suppliers' recommended protocol or validation profile for the given application unless otherwise stated.

### Generation of constructs

Constructs were generated by standard cloning techniques. Briefly, PCR was carried out using Q5 High Fidelity Hot Start DNA Polymerase (New England Biolabs; NEB), using 20 pmol primers and 200 pg template DNA, as per manufacturers' instructions. PCR products were purified using QIAquick PCR Purification Kit (QIAgen). Restriction digest reactions were carried out using NEB restriction enzymes at 37°C for 45–60 min. Ligation reactions were carried out using T4 DNA Ligase (NEB) overnight at 16°C. Transformation was carried out in *Escherichia coli* cells (α-select, XL1-Blue, or BL21-DE3 cells were used, depending on the application; all originally sourced from NEB) for 30 min on ice, followed by heat shocking (45 s, 42°C), and a further 15 min on ice. Cells were recovered by incubating in LB medium at 37°C for 1 h and then plated on LB-agar plates containing appropriate antibiotics. Plasmids were prepared by mini or maxi preps using commercially available kits (Qiagen and Promega, respectively) following the manufacturer's instructions, and verified by DNA sequencing using Eurofins Genomics TubeSeq service.

Specifically, human Tau 4R0N isoform WT or P301L coding sequences were amplified from pSinRep5-EGFP-Tau-expressing plasmids (a gift from Prof. Neil Marrion, University of Bristol, UK) by PCR and cloned with an N-terminal Myc-tag into the SpeI and BamHI sites of the lentiviral plasmid pXLG3-PX-WPRE (Wilkinson et al., 2022).

All plasmid sequences and DNA are available upon request from corresponding authors.

### Protein purification

Proteins were purified for use in MitoLuc import assays, as detailed previously (Needs et al., 2023). Protein expression was carried out as described previously (Pereira et al., 2019). A single colony of transformed BL21 (DE3) bacteria was grown in LB medium with appropriate antibiotic overnight (37°C; 200 rpm). Pre-cultures were used to inoculate a secondary culture at a 1:100 dilution, in 2× yeast extract tryptone (YT) supplemented with appropriate antibiotics. Secondary cultures were grown until mid-log phase, then induced with 1 mM IPTG or 0.2% (w/v) arabinose and grown for a further 3 h. Cells were harvested by centrifugation (15 min; 6000 ***g***), resuspended in TK buffer (20 mM TRIS base, 50 mM KCl; pH 8.0), cracked in a cell disruptor (Constant Systems; 2 cycles at 25 kpsi), and clarified by centrifugation (45 min; 103,500 **g**).

#### GST-tagged recombinant perfringolysin O

Recombinant perfringolysin O (rPFO) was used to permeabilise cell membranes in MitoLuc import assays, as described previously (Needs et al., 2023). Supernatant from the protein expression (as described above) (soluble fraction) was loaded onto a 5 ml GSTrap 4B column (GE Healthcare) and the column was washed with TK buffer until the absorbance of the flow through ceased to decrease any further. The peptide was eluted using 10 µM reduced glutathione, prepared fresh. Eluted fractions were pooled and loaded onto a 5 ml anionic exchanger (Q-column; GE Healthcare). A salt gradient of 0–1 M was applied over 20 min and the protein was eluted in 5 ml fractions. The fractions containing the protein were confirmed by SDS-PAGE with Coomassie staining, then pooled and spin concentrated. The final protein concentration was determined based on an extinction coefficient of 117,120 M^−1^ cm^−1^. The protein was aliquoted, snap-frozen and stored at −80°C until required. For each assay, a fresh aliquot was thawed, used immediately, and discarded afterwards, due to the instability of this protein (Lei and Bochner, 2021).

#### GST-Dark peptide

GST-Dark peptide was used to quench the background signal in MitoLuc import assays, as described previously (Needs et al., 2023). GST–Dark was prepared as described previously (Pereira et al., 2019). The supernatant from the protein expression (as described above) was loaded onto a GSTrap 4B column and purification was carried out exactly as for rPFO but without the ion exchange chromatography. Analysis, yield, and freezing were carried out exactly as for rPFO. Protein concentration was determined based on an extinction coefficient of 48,360 M^−1^ cm^−1^.

#### His-tagged Su9-EGFP-pep86

Su9-EGFP-pep86 was used as a precursor protein to monitor import efficiency in MitoLuc import assays, as described previously (Needs et al., 2023). Inclusion bodies from the protein expression (as described above) (insoluble fraction) were solubilised in TK buffer supplemented with 6 M urea, before loading onto a 5 ml HisTrap HP column (GE Healthcare). The protein was eluted in 300 mM imidazole. Eluted fractions containing the desired protein were pooled and loaded onto a 5 ml cationic exchanger (S- column; GE Healthcare). A salt gradient of 0–1 M was applied over 20 min and the protein was eluted in 5 ml fractions. Excess salt was removed by spin concentration, followed by dilution in TK buffer containing 6 M urea. Analysis, yield, and freezing were carried out exactly as for rPFO. Protein concentration was determined based on an extinction coefficient of 28,880 M^−1^ cm^−1^.

### Cell culture

#### HeLa and HEK293T cell culture

HEK293T (from the ECACC) and glucose-grown HeLa cells (HeLaGLU; from the ATCC) were maintained in Dulbecco's modified Eagle's medium (DMEM; Gibco; 41965039) supplemented with 10% (v/v) fetal bovine calf serum (FBS; Invitrogen) and 1% (v/v) penicillin-streptomycin (P/S; Invitrogen). When OXPHOS dependence was required, HeLa cells were cultured in galactose medium (HeLaGAL; ATCC) consisting of DMEM without glucose (Gibco; 11966025) supplemented with 10 mM galactose, 1 mM sodium pyruvate, 10% FBS and 1% P/S. Cells were cultured in galactose medium for at least 3 weeks before experiments on HeLaGAL cells. Cells were maintained in T75 ventilated flasks in humidified incubators at 37°C with 5% CO_2_. Cell lines were regularly mycoplasma tested using Eurofins mycoplasma testing service.

#### Primary neuronal cell culture

Primary neuronal culture was carried out following established lab protocols, as described previously (Martin and Henley, 2004). All animal care and procedures were carried out in full compliance with University of Bristol and ARRIVE guidelines, and the UK Animals Scientific Procedures Act, 1986. In addition, all experimental protocols were approved by University of Bristol Animal Welfare and Ethics Review Body (ethics approval number UIN: UB/18/004) panel and the Biological and Genetic Modification Safety Committee (BGMSC). Briefly, primary hippocampal and cortical neurons were isolated from embryonic day 18 (E18) Han Wistar rat pups. Pregnant Han Wistar rats, bred in-house at the University of Bristol Animal Services Unit, were anaesthetised using isoflurane with pure oxygen flow and humanely killed by means of cervical dislocation, following Home Office Schedule 1 regulations. Isolated cortices and hippocampi were washed extensively in HBSS and dissociated by incubation with 10% (v/v) Trypsin-EDTA solution at 37°C for 15 and 9 min, respectively. Cells were grown in plating medium [Neurobasal medium (Gibco) supplemented with 5% (v/v) horse serum (Sigma), 2% (v/v) B27 (Gibco), 1% P/S, and 5 mM Glutamax (Gibco)]. After 3 h, plating medium was removed and replaced with feeding medium (Neurobasal medium supplemented with B27, P/S, and 2 mM Glutamax). For biochemistry, cortical neurons were seeded at a density of 500,000 cells per well and, for imaging experiments, hippocampal neurons were seeded at a density of 150,000 cells per well, where one well represents a ∼35 mm surface. All plates (containing sterile coverslips if appropriate) were incubated with poly-L-lysine [0.5 mg/ml or 1 mg/ml for plastic or glass, respectively, in sterile borate buffer (1 mM borax and 5 mM boric acid)] overnight, extensively washed with tissue culture grade H_2_O and incubated overnight in plating medium before plating cells, to aid adhesion. Cells were maintained in humidified incubators at 37°C with 5% CO_2_.

### Transfection of cells

#### HeLa cell transfection

HeLa cells were plated and grown up to ∼70–80% confluency. At this point cells were transfected with 1 μg (per 35 mm dish) of the desired DNA using Lipofectamine 3000 reagent (Thermo Fisher Scientific), at a 1:1.5 ratio of DNA:Lipofectamine, following the manufacturer's protocol. Cells were then grown for a further 24–72 h before experimental analysis.

#### Primary neuron transfection

Primary neurons were transfected at 14 days *in vitro* (DIV14) with 0.5–1 μg of the desired DNA (per 35 mm dish) using Lipofectamine 2000 reagent (Thermo Fisher Scientific), at a ratio of 1.5 µl Lipofectamine 2000 per 1 µg DNA. Coverslips with adhered neurons were washed in plain neurobasal medium and transferred to dishes containing 1 ml plain neurobasal medium, to which the transfection mix was added. At 45 min after transfection, coverslips were washed again to remove all remaining DNA and Lipofectamine 2000 from cells and returned to their conditioned growth medium. Neurons were then maintained for a further 7 days before fixation.

### Cell transduction by lentiviral infection

Lentiviral particles were produced in HEK293T cells by the addition of a mixture of DNAs [27.2 µg DNA to be produced, and packaging vectors pMDG2 (6.8 µg) and pAX2 (20.4 µg)] and pEI transfection reagent (1.5:1 pEI:DNA to be produced) in OptiMEM medium (Gibco) to HEK293T cells in a T75 flask, followed by incubation for 6 h at 37°C, 5% CO_2_. Medium was then changed to complete DMEM, and cells were incubated for 72 h to allow lentivirus particle production. Lentivirus particles were harvested at 48 and 72 h for maximum yield, pooled, spun down at 4000 ***g*** for 5 min to remove dead cells, and concentrated by adding Lenti-X concentrator (Takara Bio) at a 1:3 ratio and incubating at 4°C for at least 1 h. Lentivirus was then pelleted by centrifugation at 4000 ***g*** for 45 min. Pellets were resuspended in plain DMEM at 1:50 of initial supernatant volume and aliquoted and stored at −80°C until required. For infection, concentrated lentivirus was added dropwise to the HeLa or neuronal cell medium, and cells were incubated before experimental analysis.

### Mitochondrial isolation

Confluent cells were harvested by trypsinisation, pelleted (200 ***g*** for 5 min) and washed extensively with HBSS. Pellets were frozen overnight at −80°C and thawed the next day, to weaken membranes. Subsequently, mitochondrial isolation was performed using Mitochondrial Isolation Kit for Cultured Cells (Abcam; ab110170) following the manufacturer's instructions. Mitochondrial protein concentration was calculated by BCA assay (Pierce™ BCA Protein Assay Kit, Thermo Fisher Scientific), following the manufacturer's instructions, and using BSA as a standard.

### Immunoprecipitation

Following mitochondrial isolation, mitochondria were gently lysed using 4.5 g glyco-diosgenin (GDN)/g protein in IP buffer [0.1 M Tris-HCl, 0.15 M NaCl, phospholipids (0.03 mg/ml phosphatidylethanolamine, 0.03 mg/ml phosphatidylglycerol, and 0.09 mg/ml phosphatidylcholine) and 1× cOmplete ULTRA Protease Inhibitor Cocktail]. Myc-tagged proteins were isolated using 10 µl Myc-Trap beads (per 10 cm dish; Chromotek). The supernatant (lysed mitochondrial sample) was incubated on a rotating wheel with beads overnight at 4°C. Subsequently, beads were washed in IP buffer. After washing, the supernatant was removed, and samples were analysed by western blotting.

### Total protein cell lysis

For extraction of total protein lysate, cells were washed extensively with HBSS, and RIPA buffer (supplemented with 1 mM PMSF) was added (200 µl for ∼35 mm surface, scaled up or down appropriately). Cells were scraped on ice into Eppendorf tubes, which were incubated for 1 h on a rotating wheel at 4°C, followed by centrifugation at 10,000 ***g*** for 15 min at 4°C. Protein samples were stored at −20°C. Protein concentration was determined through a BCA assay.

### Western blotting

Following protein extraction, samples were heated to 95°C for 5 min in the presence of lithium dodecyl sulfate (LDS) sample buffer supplemented with 50 mM DTT. 30 μg of total protein was loaded on 4–12% BOLT gels (Thermo Fisher Scientific), separated (200 V, 24 min), and transferred onto polyvinylidene difluoride (PVDF) membranes (activated with methanol; Thermo Fisher Scientific) with transfer buffer (336 mM Tris, 260 mM glycine, 140 mM tricine, 2.5 mM EDTA) using a semi-dry Pierce Power Station transfer system (Thermo Fisher Scientific; 2.5 mAmp, 10 min). Membranes were blocked for 1 h in milk [5% (w/v) in TBS-T; 20 mM Tris-HCl, 1.5 M NaCl and 0.1% (v/v) Tween-20 (pH 7.6)] and incubated in 5% milk containing the appropriate primary antibody (4°C, overnight; see figure legends). Membranes were washed extensively with TBS-T and probed with the appropriate secondary antibody in 2.5% milk [room temperature (RT), 1 h]. Membranes were washed with TBS-T, incubated with ECL substrate (GE Healthcare) and developed using an Odyssey Fc Imaging System (LI-COR). Analysis and quantification were carried out using Image Studio Lite software. For transparency, uncropped blots are shown in Fig. S4.

### MitoLuc assay

MitoLuc assays were carried out exactly as described previously (Needs et al., 2023). Briefly, cells expressing *eqFP670-P2A-Cox8a-11S* and GFP, Tau^WT^ or Tau^P301L^ were plated on standard white flat-bottom 96-well plates. Where appropriate, cells were treated with 50 nM cytochalasin D, 100 nM nocodazole, or DMSO only for 48 h prior to assays. On the day of the assay, cells were washed with HBSS and incubated in HBSS wash buffer [HBSS supplemented with 5 mM D-(+)-glucose, 10 mM HEPES (Santa Cruz Biotechnology, Germany), 1 mM MgCl_2_, 1 mM CaCl_2_; pH 7.4]. A fluorescence read for normalisation to eqFP670 levels was taken at 605/670 nm using monochromators with the gain set to allow maximum sensitivity without saturation, using a BioTek Synergy Neo2 plate reader. Cells were transferred to permeabilised cell assay master mix (225 mM mannitol, 10 mM HEPES, 2.5 mM MgCl_2_, 40 mM KCl, 2.5 mM KH_2_PO_4_, 0.5 mM EGTA; pH 7.4) supplemented with 5 mM succinate, 1 µM rotenone, 0.1 mg/ml creatine kinase, 5 mM creatine phosphate, 1 mM ATP, 0.1% (v/v) Prionex, 3 nM rPFO (purified in house), 20 µM GST-Dark (purified in-house) and 1:800 furimazine (Nano-Glo^®^ Luciferase Assay System; Promega). A baseline read of 30 s of background luminescent signal was taken before injection of the purified substrate protein (*Su9-EGFP-pep86*; purified in house) to 1 µM final concentration, followed by a further bioluminescence read corresponding to import, lasting 30 min. Bioluminescence was read using a BioTek Synergy Neo2 plate reader (Agilent, UK) or a CLARIOstar Plus plate reader (BMG LabTech, UK) without emission filters with the gain set to allow maximum sensitivity without saturation, and with an acquisition time of 0.1 s per well. Row mode was used, and reads were taken with the minimum interval, with wells in triplicate.

### Confocal microscopy

#### Fixed-cell confocal microscopy

Cells on glass coverslips were washed in HBSS and fixed in 4% (v/v) paraformaldehyde (PFA) with 2% (w/v) sucrose (sucrose used for neurons only, to retain osmolarity), for 12 min at 37°C. Coverslips were then washed extensively in HBSS, and PFA was quenched using 100 mM glycine. Cells were then stained for immunocytochemistry or mounted.

For wheat germ agglutin (WGA) staining, cells were incubated with 5 µg/ml (WGA) Alexa Fluor 633 (Thermo Fisher Scientific) for 10 min at RT then washed three times in HBSS.

For immunocytochemistry, fixed cells were permeabilised by incubation in 0.1% (v/v) Triton-X in PBS for 5 min, washed in PBS and blocked in 3% (w/v) BSA in PBS for 30 min. Coverslips were incubated on a drop of primary antibody in 3% BSA overnight at 4°C. Coverslips were washed extensively in PBS and incubated with the appropriate secondary antibody (2 h, RT) in 3% BSA, then washed again.

Coverslips were dipped in ddH_2_O and mounted onto slides using Fluoromount-G mounting medium (Thermo Fisher Scientific) with or without DAPI. Coverslips were left to dry overnight and imaged using a Leica confocal microscope (SP5II or SP8) and the LAS X software platform. Laser lines used were 405, 488, 562 and 633 nm, with the gain set to allow maximum sensitivity without saturation, and *z*-stacks were taken.

#### Live-cell confocal microscopy

Transfected cells were grown to confluency on 35 mm glass-bottomed dishes (Corning). Cells were washed in HBSS and incubated in HBSS imaging buffer [HBSS supplemented with 5 mM glucose, 10 mM HEPES, 1 mM MgCl_2_, 1.26 mM CaCl_2_] with 25 nM tetramethylrhodamine (TMRM) for 30 min at 37°C. Cells were imaged immediately, with TMRM retained in the buffer, using a Leica SP8 confocal microscope and LAS X software platform, at 37°C.

### Image analysis

All image analysis was performed using the FiJi image processing package (Rueden et al., 2017; Schindelin et al., 2012). Macros were written by Dr Stephen Cross (Wolfson Bioimaging Facility, University of Bristol, UK), within the FiJi plugin Modular Image Analysis (MIA) package version 0.21.0, which is publicly accessible on GitHub with a linked version-specific DOI from Zenodo (Cross et al., 2023 preprint). Where data were analysed manually, data were anonymised first.

#### Mitochondrial morphology analysis

Mitochondrial pre-processing, as well as branch and network analysis, was carried out as described previously (Cross and Seager, 2022; Seager, 2020), using a FiJi plugin adapted from the mitochondrial network analysis (MiNA) toolset (Valente et al., 2017). Briefly, for pre-processing, cells of interest were outlined, and the outside was cleared, then a *z*-stack maximum intensity projection was performed, followed by contrast enhancement and background subtraction. Median, unsharp and tubeness filters were applied. For branching and network analysis, images were binarised and skeletonised, and the skeleton was analysed to obtain information on the branches and pixels, allowing analysis of mitochondrial branching as a readout of mitochondrial network complexity and fragmentation.

#### Membrane potential analysis

TMRM analysis of mitochondrial membrane potential was performed based on a published protocol (Creed and McKenzie, 2019). Briefly, an average of the first ten frames (TMRM fluorescence where images were captured by live-cell confocal microscopy over 2 min) was taken, and then the mitochondria were thresholded and their intensity measured. The average of the final three frames [after carbonyl cyanide m-chlorophenylhydrazone (CCCP) treatment to dissipate the ΔΨ] was subtracted as a control and the intensity of the subtracted image was re-measured.

#### Neuronal morphology analysis

Analysis of neuronal complexity (number of processes, i.e. axon and dendrites) was carried out by manually counting the number of processes protruding from the soma (at a given distance from the soma – highlighted by circles on images). This was carried out on anonymised data, where expression of the protein of interest had been confirmed before data anonymisation, and all processes were highlighted using a mCherry cytosolic marker.

Initial segments of axons in transfected cells were identified by staining for ankyrin-G. Following the identification of the axon to be measured, the length of the axon was measured by tracing the length of the axon, highlighted by the mCherry cytosolic marker, using the Simple Neurite Tracer (SNT) component of the Neuroanatomy plugin in FiJi.

Synapses were counted manually. First, data was anonymised, and dendrites were identified. The dendrite of interest was measured using the SNT plugin and the synapses along the region of interest were counted manually using the PSD95 stain as reference. A total of five dendrites were analysed per cell and an average was taken for each cell.

### Seahorse assays – mitochondrial stress test

Cells were seeded in six-well plates (200,000 cells/well) and transfected appropriately, as described in figure legends. The day before the assay, cells were detached by trypsinisation, counted, and seeded at 10,000 cells/well in 96-well Seahorse XF cell culture plates (Agilent). The sensor cartridges were hydrated overnight with tissue culture grade H_2_O in a non-CO_2_ incubator at 37°C as per the manufacturer's instructions. On the day of the assay, H_2_O in the sensor plate was replaced with Seahorse XF Calibrant (Agilent) and cells were washed with HBSS and incubated in Seahorse medium [Seahorse XF assay medium (Agilent), 1 mM pyruvate (Agilent), 2 mM glutamine (Agilent) and 10 mM D-(+)-galactose]. Both sensor and cell plates were incubated in a non-CO_2_ incubator at 37°C for 1 h before the assay. The sensor plate was loaded with oligomycin (15 µM for 1.5 µM final concentration in wells; injector A), CCCP (5 µM for 0.5 µM final; injector B), and antimycin A and rotenone (5 µM/5 µM for 0.5 µM/0.5 µM final; injector C). The sensor plate was calibrated before loading cells and running a mitochondrial stress test using a Seahorse XFe96 (Agilent). Following assays, cells were washed and fixed in 1% (v/v) acetic acid in methanol at −20°C overnight for sulforhodamine B (SRB) assays, which were used for normalising data to protein content.

### SRB assay

Cells were fixed with ice-cold 1% acetic acid in methanol overnight at −20°C. Fixative was aspirated and plates were allowed to dry at 37°C. SRB [0.5% (w/v) in 1% (v/v) acetic acid in dH_2_O] was added to cover cells, and plates were incubated for 30 min at 37°C. SRB was then aspirated, and any unbound stain was removed by washing the plates four times with 1% acetic acid in dH_2_O, before drying plates at 37°C. The bound protein stain was solubilised by shaking incubation with 10 mM Tris-HCl (pH 10; 15 min, RT). Absorbance was read on a microplate reader with a 544/15 nm filter.

### DHFR-MTX precursor trapping

For precursor trapping experiments, the DHFR–MTX affinity system was utilised, as has been characterised previously (Chacinska et al., 2003; Eilers and Schatz, 1986; Ford et al., 2022; Needs et al., 2022 preprint; Rassow et al., 1989). DIV14 neuronal cells were pre-treated with 100 nM MTX by adding it dropwise to the cell medium the night before transduction. The next day, cells were subjected to the over-production of Su9-EGFP–DHFR either by transfection or infection (as detailed in the main text) and cells were incubated for a further 7 days. At DIV21, cells were fixed (for imaging) or lysed (for western blotting) for further experimental analysis. Cells expressing Su9-EGFP–DHFR without MTX are used as a control for the effects of trapping, and cells expressing EGFP–DHFR (no MTS) with or without MTX are used to control for non-trapping related effects of MTX.

### Statistical analysis

The sample size was determined based on previous research. Sample size (*N*) for all experiments was 3–6 biological replicates, with technical replicates or individual objects (cells/mitochondria) analysed for each biological replicate, as appropriate. Statistics were always performed on biological replicates. Normality of distribution was first tested, and statistical significance between groups was determined using an unpaired two-tailed Student's *t*-test or one or two-way ANOVA with interaction if more than two groups were analysed. ANOVA was nested if multiple objects were analysed per biological replicate. Following ANOVA, *P*-values were adjusted for multiple comparisons through Tukey's or Šidák's post hoc tests and differences were considered significant at a 5% level. Statistical analyses were performed using Graph Pad Prism version 9 (GraphPad Software, Inc., San Diego, CA, USA). Error bars on graphs always represent the standard deviation of the mean (mean±s.d.). Asterisks represent significance as follows: ns=*P*>0.05, *P≤0.05, **P≤0.01, ***P≤0.001, ****P≤0.0001. Individual points (for each biological replicate or object where appropriate, as detailed in the figure legend) are graphed to show the true spread of the data.

## Supplementary Material



10.1242/joces.260993_sup1 Supplementary information
