# Supplementary material for: Aggregation-prone Tau impairs mitochondrial import, which affects organelle morphology and neuronal complexity
Source: J Cell Sci. 2023 Jul 7;136(13):jcs260993. doi: 10.1242/jcs.260993 (PMC10357015; doi:10.1242/jcs.260993)
Supplement: Supplementary information [file joces-136-260993-s1.pdf]

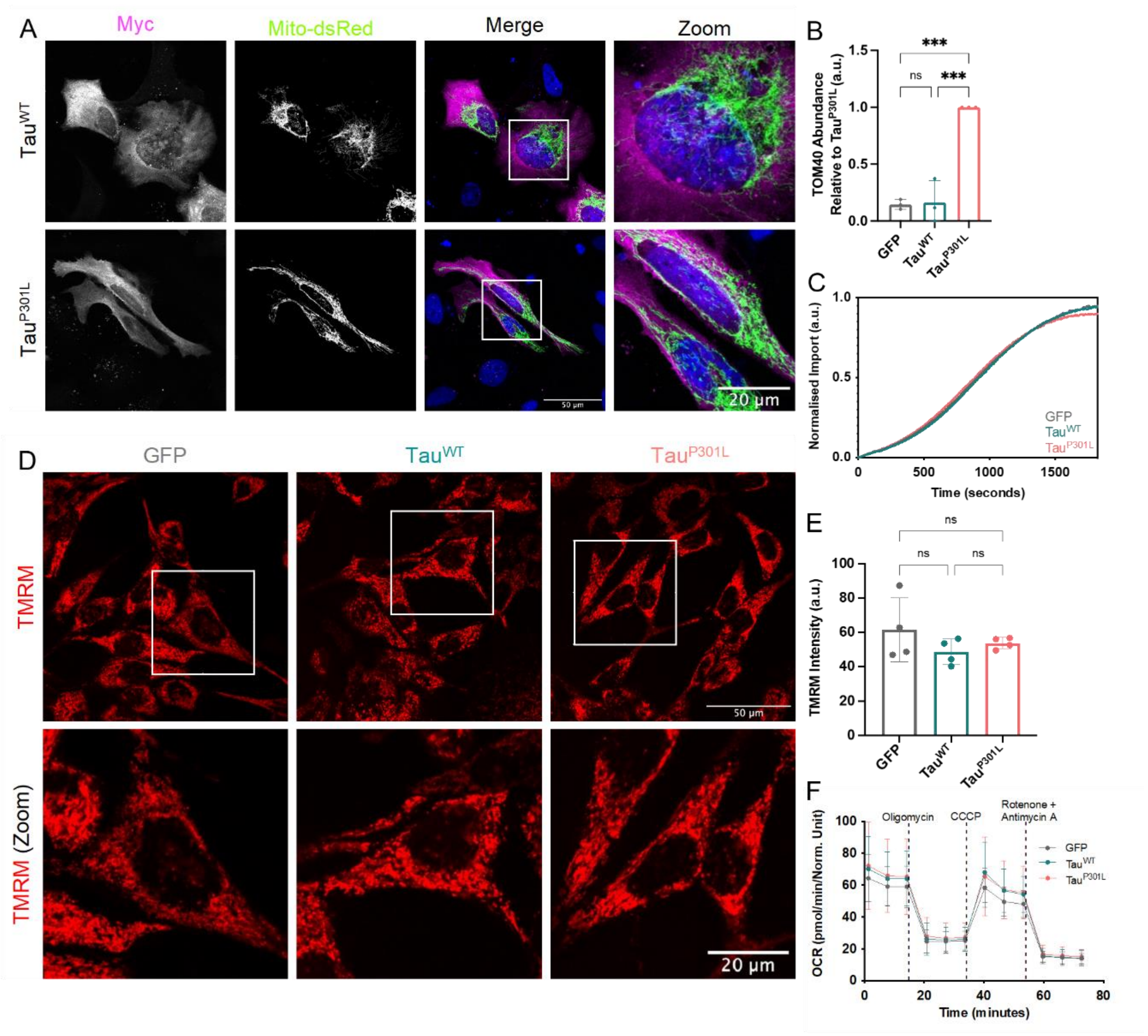

Fig. S1. (A) Representative confocal images showing mitochondria within HeLaGAL cells subjected to the over-production of mito-dsRed (green; to indicate mitochondrial localisation) and Myc-Tau^{WT} or Myc-Tau^{P301L} for 48 h. Cells were fixed and stained against Myc (CST 9B11; magenta; 1:1000) to indicate Tau localisation, and DAPI (blue; nuclei). N=4 biological replicates. **(B)** Quantification of the immunoprecipitation data in Fig. 1D, showing the abundance of TOM40 in the pulldown elution after immunoprecipitation of Myc-GFP, Myc-Tau^{WT}, or Myc-Tau^{P301L} from the mitochondrial fraction of HeLaGAL cells. The relative abundance was obtained by normalising levels in the GFP and Tau^{WT} samples to levels in the Tau^{P301L} sample. N=3 biological replicates. One-way ANOVA and Tukey's *post hoc* test were used to determine significance. Error bars show SD. P values (L-R, bottom-top): 0.9807, 0.0002, 0.0002. Representative Western blots are shown in Fig. 1D. **(C)** MitoLuc import assay trace showing the import of precursor protein *Su9-EGFP-pep86* in HeLaGAL cells over-producing *Cox8a-11S* and GFP, Tau^{WT}, or Tau^{P301L}. Averaged,

Journal of Cell Science • Supplementary information

normalised traces are shown (normalised to eqFP670 expression and max amplitude/run), and error bars represent SD. N=3 biological repeats were performed. This trace corresponds to the quantification of import amplitude shown in Fig. 1E. **(D)** Representative confocal images (left) showing TMRM fluorescence (red) in the mitochondria of HeLaGAL cells over-producing Myc-GFP, Myc-Tau^{WT}, or Myc-Tau^{P301L}. CCCP was added after 2 min to control for background fluorescence (not shown). N=4 biological replicates. **(E)** TMRM intensity was quantified (right) using a FiJi macro (see Methods). Error bars show the SD. One-way ANOVA and Tukey's *post hoc* test were used to determine significance. P values (L-R, bottom-top): 0.3212, 0.8181, 0.6398. **(F)** Mitochondrial stress test showing the oxygen consumption rate (OCR) of HeLaGAL cells over-producing Myc-GFP, Myc-Tau^{WT}, or Myc-Tau^{P301L}. Data are normalised to the protein content of each well as determined by SRB assays. Error bars show the SD. N=6 biological replicates, each with 3 technical replicates.

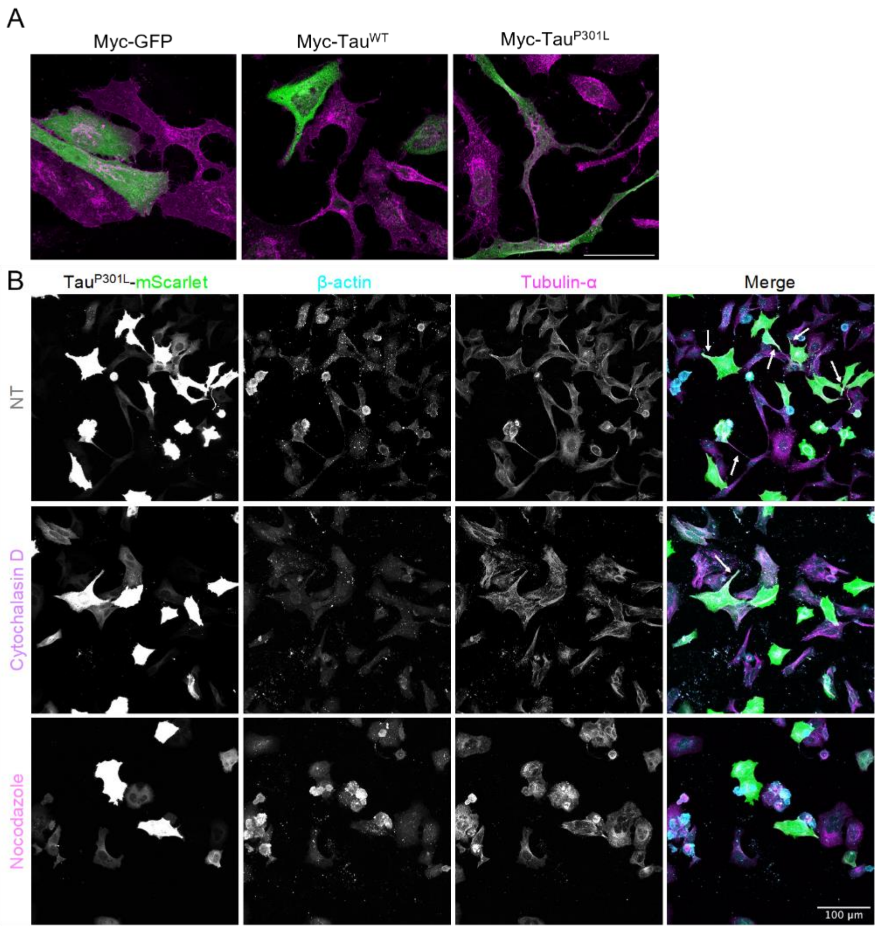

Fig. S2. (A) Representative confocal images showing the cell morphology of HeLaGAL cells subjected to over-production of Myc-GFP, Myc-Tau^{WT}, or Myc-Tau^{P301L} for 48 h. Cells were fixed and stained with Wheat Germ Agglutin (WGA; magenta), a membrane stain, to highlight TNTs. Co-staining was carried out against Myc (CST 9B11; green; 1:1000) to indicate TNT formation between transfected and untransfected cells. N=4 biological replicates. **(B)** Representative confocal images of HeLaGAL cells exposed to Tau^{P301L} over-production in the presence of TNT inhibitors. HeLaGAL cells were subjected to over-production of Tau^{P301L}-mScarlet (green) and either untreated (NT; DMSO only) or treated with 50 nM Cytochalasin D or 100 nM Nocodazole (48 h). Cells were fixed and stained for β-actin (cyan; 1:1000) and tubulin-α (magenta; BioRad MCA78G; 1:500). Arrows in merge highlight TNTs. N=3 biological replicates. Quantification is shown in Fig. 2D.

Journal of Cell Science • Supplementary information

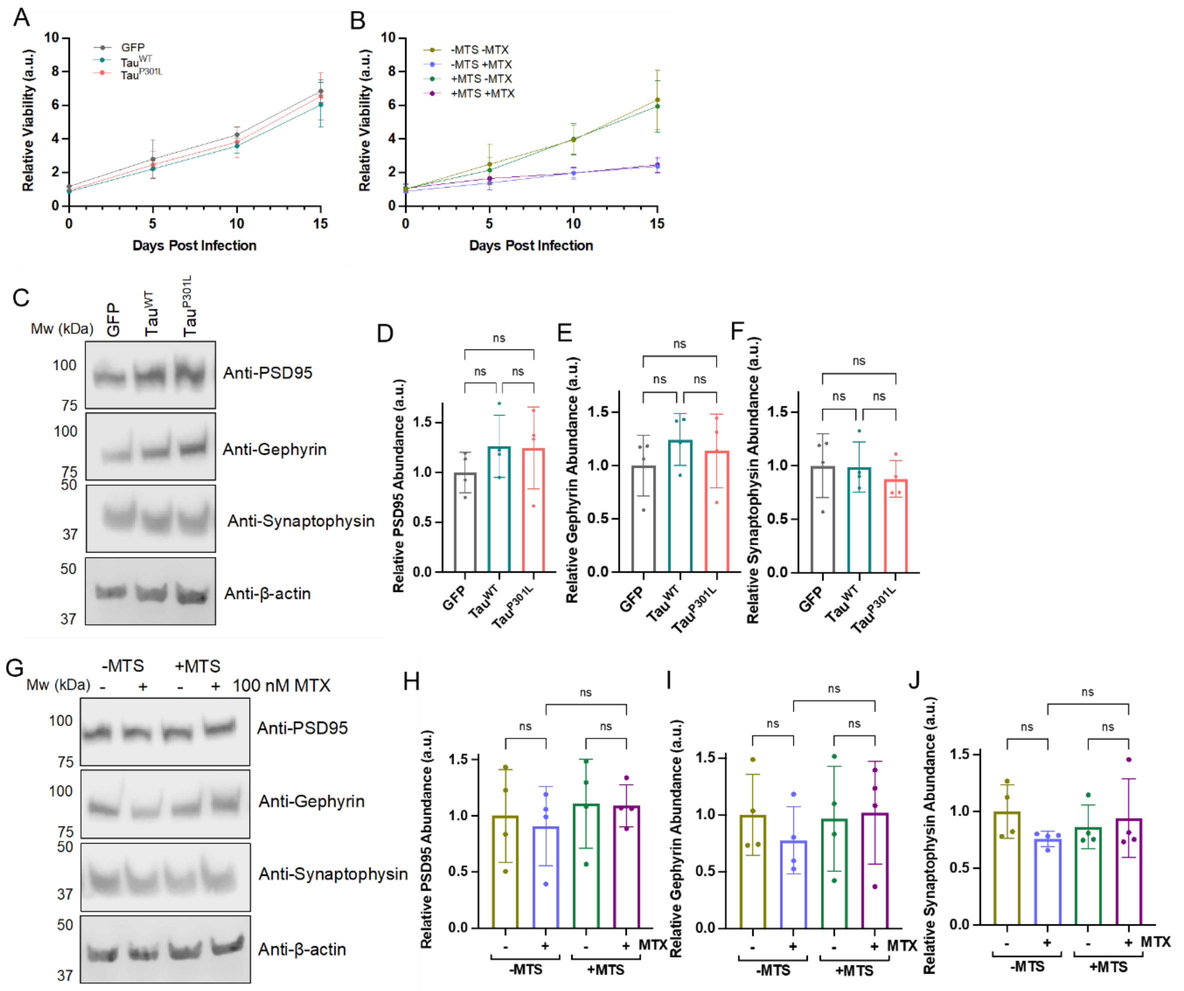

Fig. S3. Viability of primary cortical neurons over-producing GFP (control), Tau^{WT} or Tau^{P301L} **(A)**, or EGFP-DHFR(-MTS) or Su9-EGFP-DHFR (+MTS) +/- 100 nM MTX **(B)**, as determined by cell density, quantified by SRB assays. Data were normalised to the average reading for the 0 days post-infection time point to give relative viability of the neurons in response to the varying expression/treatment. N=4 biological replicates. Error bars show SD*.* **(C)** Representative Western blots showing the levels of PSD95 (1:1000), Gephyrin (Synaptic Systems; 147111; 1:1000), and Synaptophysin (Merck Millipore; 573822; 1:1000) in DIV21 cortical neurons after 7 days of over-production of Myc-GFP, Myc-Tau^{WT}, or Myc-Tau^{P301L}. β-actin was used as a loading control. N=4 biological replicates. Histograms show quantification of PSD95 **(D)**, Gephyrin **(E)**, and Synaptophysin **(F)** relative abundance, normalised to β-actin and relative to levels in the GFP control sample. Error bars show SD. One-way ANOVA and Tukey's *post hoc* test were used to determine significance. P values (L-R, bottom-top): PSD95: 0.5076, 0.9979, 0.5422; Gephyrin: 0.4992, 0.8688, 0.7917; Synaptophysin: 0.9981, 0.7904, 0.7581. **(G)** Representative Western blots

showing the levels of PSD95, Gephyrin, and Synaptophysin in DIV21 cortical neurons after 7 days of over-production of EGFP-DHFR (-MTS) or Su9-EGFP-DHFR (+MTS) +/- 100 nM MTX. β-actin was used as a loading control. N=4 biological replicates. Histograms show the quantification of PSD95 **(H)**, Gephyrin **(I)**, and Synaptophysin **(J)** relative abundance, normalised to β-actin and relative to levels in the -MTS -MTX control sample. Error bars show SD. One-way ANOVA and Tukey's *post hoc* test were used to determine significance. P values (L-R, bottom-top): PSD95: 0.9810, 0.9998, 0.8782; Gephyrin: 0.8560, 0.9974, 0.8216; Synaptophysin: 0.4862, 0.9662, 0.6959.

Journal of Cell Science • Supplementary information

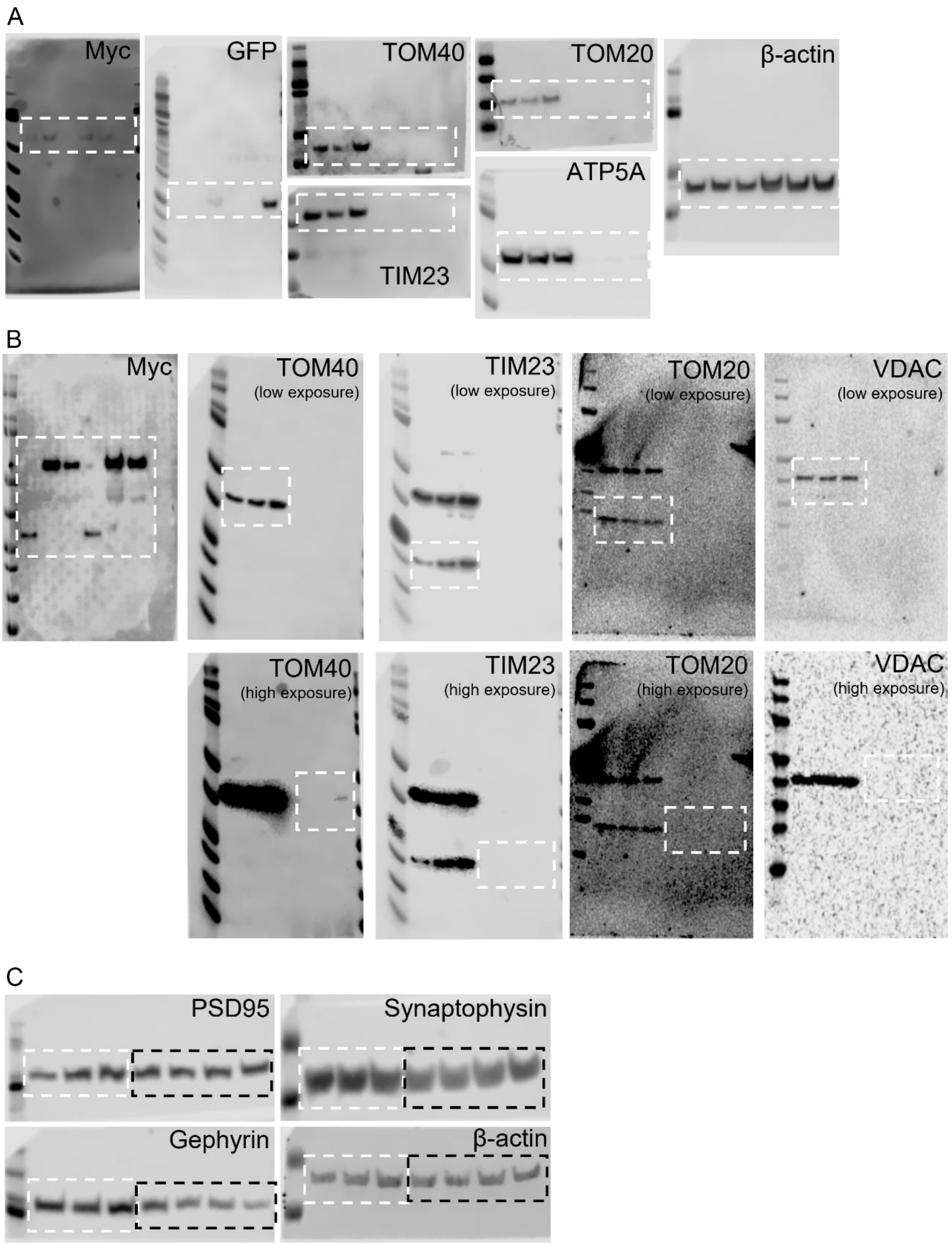

Fig. S4. Uncropped representative Western blots from Fig. 1A **(A)**, Fig. 1D **(B)**, and Fig. S3C and G **(C)**. The box shows the area cropped in the main figure. For Western blots in Fig. 1D where multiple exposures were shown in one figure, both exposures have been indicated in **(B)**. Western blots in Fig. S3C and G were performed concurrently on the same membrane, and therefore are shown together; the white box shows the area cropped in Fig. S3C, and the black box shows the area cropped in Fig. S3G.

Journal of Cell Science • Supplementary information
