# Supplementary material for: Aggregation-prone Tau impairs mitochondrial import, which affects organelle morphology and neuronal complexity
Source: J Cell Sci. 2023 Jul 7;136(13):jcs260993. doi: 10.1242/jcs.260993 (PMC10357015; doi:10.1242/jcs.260993)
Supplement: Supplementary file 1 [file joces-136-260993_review_history.pdf]

Aggregation-prone Tau impairs mitochondrial import, which affects organelle morphology and neuronal complexity

Hope I. Needs, Kevin Wilkinson, Jeremy Henley and Ian Collinson
DOI: 10.1242/jcs.260993

Editor: Giampietro Schiavo

Review timeline

Original submission:	18 January 2023
Editorial decision:	13 February 2023
First revision received:	17 May 2023
Editorial decision:	26 May 2023
Second revision received:	26 May 2023
Accepted:	5 June 2023

Original submission

First decision letter

MS ID#: JOCES/2023/260993

MS TITLE: Perturbation of mitochondrial import by pathogenic Tau affects organelle morphology and reduces neuronal complexity

AUTHORS: Hope I. Needs, Kevin Wilkinson, Jeremy Henley, and Ian Collinson
ARTICLE TYPE: Research Article

We have now reached a decision on the above manuscript.

To see the reviewers' reports and a copy of this decision letter, please go to: https://submit-jcs.biologists.org and click on the 'Manuscripts with Decisions' queue in the Author Area. (Corresponding author only has access to reviews.)

As you will see, the reviewers raise a number of substantial criticisms highlighting concerns about several aspects of your experimental approach. These currently prevent me from accepting the paper at this stage. They suggest, however, that a fully revised version might prove acceptable, if you can address their concerns. This would need a significant amount of experimental and editorial work. If you think that you can deal satisfactorily with the criticisms on revision, I would be pleased to see a revised manuscript. We would then return it to the reviewers.

Please ensure that you clearly highlight all changes made in the revised manuscript. Please avoid using 'Tracked changes' in Word files as these are lost in PDF conversion.

I should be grateful if you would also provide a point-by-point response detailing how you have dealt with the points raised by the reviewers in the 'Response to Reviewers' box. Please attend to all of the reviewers' comments. If you do not agree with any of their criticisms or suggestions please explain clearly why this is so.

© 2023. Published by The Company of Biologists under the terms of the Creative Commons Attribution License (https://creativecommons.org/licenses/by/4.0/).

Reviewer 1

Advance summary and potential significance to field

Needs et al. have investigated the impact of pathogenic Tau mutant (TauP301L) on mitochondrial function and neuronal complexity. This is an important question and of broad relevance. The authors propose that TauP301L specifically interacts with the mitochondrial import machinery, which mildly disturbs mitochondrial morphology but does not impair mitochondrial import efficiency or OXPHOS. They observe tunnelling nanotubes (TNTs) in HeLa cells expressing TauP301L and suggest that organelle exchange via TNTs is required to maintain mitochondrial quality and import efficiency. Primary hippocampal neurons expressing TauP301L have fewer processes and dendritic synapses, which the authors attribute to defective mitochondrial import since they observe similar effects in neurons with artificially blocked mitochondrial import.

Previous studies have described various impacts of Tau on mitochondrial function and it is indeed interesting to consider the deleterious effect that mutant Tau may have on mitochondrial import efficiency. However, in my opinion the authors do not currently provide evidence to support their key claims that mutant Tau can disturb import or that this is the reason for reduced neuronal complexity.

Comments for the author
Main points

In Fig. 1, the authors blot TauP301L in mitochondrial fractions and identify an interaction with TOM40 by IP. There is no effect on mitochondrial import (S2) or oxygen consumption (S3) in TauP301L expressing cells. I would argue that any interaction between TauP301L and TOM40 does not necessarily mean that pathogenic Tau perturbs mitochondrial import, which is the central message of the manuscript. In Fig. 2, the authors present evidence of TNT formation and state that these can likely rescue import deficiencies in TauP301L expressing cells. A key experiment would be to perform import assays in TauP301L expressing cells that are unable to form TNTs (e.g as performed in the recent Needs et al., 2022 with the DHFR + MTX assay and nocodazole). This is required to test whether TauP301L can block import.

Unfortunately, similar issues arise in the primary neuron work. The effects of TauP301L on neuronal complexity are entirely correlative with the artificial import blocker experiments, which the authors assume to be evidence of similar consequence to perturbed mitochondrial import, which is not shown in these cells. I appreciate it would be difficult to abolish TauP301L interaction with TOM40 in neurons, without knowing what drives this interaction, but this would help argue that the interaction between TauP301L and TOM40 is relevant to neuronal complexity.
Other points

- The interaction between TauP301L and TOM40 is also not entirely convincing as presented. Is the IP in Fig1D on a single membrane (as shown for controls in Fig S1) and what is the % input? What is the evidence that the TOM40 interaction is specific as the authors claim, apart from not interacting with TOM20 and TIM23 (S1)? Furthermore, can the authors also comment on the minimal colocalization between TauP301L and mitochondria in Fig 2C – confocal colocalization of TauP301L and the mitochondrial network would support the fractionation data and may give further clues to the inclusion of TauP301L decorated mitochondria in TNTs.
- The same cell with mito-dsRed appears to have been used for images in Fig 2C and Fig. 1F. The authors should mention this.
- Are stats performed on “biological replicates” (as I think should be) or individual cells e.g in Fig 1G legend there are two definitions of the N number?

Reviewer 2

Advance summary and potential significance to field

The study by Needs and colleagues explores mitochondrial protein-import dysfunction as a cause of neurodegeneration by exploiting a Tau model of neurodegeneration. The authors suggest:

© 2023. Published by The Company of Biologists under the terms of the Creative Commons Attribution License (https://creativecommons.org/licenses/by/4.0/).

- a Tau mutant - TauP301L binds to the protein-channel of the outer-membrane TOM40
- the interaction between TauP301L and Tom40 affects mitochondrial morphology, but does NOT impact mitochondrial import, or respiratory function
- TauP301L induces the formation of tunneling nanotubes (TNTs)
- primary neuronal cultures show morphological changes characteristic of neurodegenerative disease in the presence of TauP301L
- changes to neuronal structure are described when mitochondrial import is blocked artificially.

Comments for the author

The authors conclude perturbations in mitochondrial protein import, specifically blockage of the central entry gate TOM complex may be an important factor in the onset of neurodegenerative diseases. This aligns with previous suggestions in the literature and is an exciting avenue of research, but unfortunately key experiments in this manuscript do not actually support this conclusion and have been over interpreted to align with this proposed model. The paper presents an exciting concept worthy of consideration, but would require significant revision to be at a standard for JOCES.

General items
[1] The title does not reflect the findings of the study. The research does not demonstrate effectively an import defect in the presence of mutant Tau. Indeed the authors show and interaction of mutant Tau with Tom40 and that import into the matrix (Fig 1E) is unaffected, themselves concluding that there is no import defect. The use of the term perturbation of protein import in the title and general conclusions of this paper is not appropriate and should be modified.

[2] The Materials and Methods are lacking clarity and information to guide the reader. The results section needs to be more explicit in some sections. For example Fig 1A, are these constructs introduced into HeLa cells using transient transfection or the lentiviral system described in the methods. The methods are lacking specific detail making it hard for the reader to comprehend what has been done where. As one example the lentiviral infection - which genes were clones into these vectors, which cells lines was this system used on? Why is there a section describing purification of GST proteins as it is not clear from the results where there was any GST proteins applied. What was the GST Darp peptide used for? There is no information on the DHFR blocking experiments and this is vital to understand what has been undertaken to come to the conclusion that there is blockage in the neurons. This methods seems to have been scrambled together and lack general QC.

[3] In general I am not comfortable with significant reference to a concurrent pre-print of research that aligns with this paper - that has yet to be peer reviewed. This is something for editorial consideration.

Comments pertaining to Data:

Figure 1
- method used for determining protein localisation % to the mitochondria is not clear. 1A should show a Total sample and how the cytosol and mitochondria compare in this regard.
- Figure 1A - Interesting that P301L expressing is impacting the levels of Tom20 and Tim23 but the protein is interacting with Tom40. Do and how do the levels of the TOM and TIM23 complexes change by BN-PAGE.
- Figure 1D is not properly controlled. Supp Figure S1 should be shown in the main Figure. These blots all need loading controls - other mitochondrial proteins to show that something like Tom40 is not coming down non-specifically. IP coupled with mass spectrometry would be a more valuable and non-biased approach to deciphering how the interaction of wild-type and mutant Tau differ.
- The panel for Tom20 (input) in Fig S1 does not align with the conclusion made in Figure 1A. In 1A levels of Tom20 are reduced in the presence of mutant Tau, while Supp S1 shows the opposite. How can the authors rationalise the conclusion made in 1A.

© 2023. Published by The Company of Biologists under the terms of the Creative Commons Attribution License (https://creativecommons.org/licenses/by/4.0/).

• it is concluded that there is an import defect (in reference to abundance of mitochondrial import subunits), but direct evidence of an import defect is lacking. Import rates may be unaffected by these small changes in subunit abundances and only one import pathway is tested with the assay in 1E (matrix import) - what about other pathways into mitochondria? If import defect is the mechanism at play this needs to be interrogated and shown with a more reliable method. For example how does the mitochondrial proteome change in the presence and absence of mutant Tau?
• There is no positive control for the NanoLuc assay. Can the readers be reassured that the assay is working?

Figure 2
• It is not clear if the whole population off cells shown here are expressing the GFP-TAU wild-type or mutant. Are these cells transiently transfected or transduced with virus and selected. If the entire population is expressing the Tau variant what purpose would TNT’s serve if all the cells are unhealthy? How could the mechanism of healthy/unhealthy mitochondrial exchange occur if there are no “healthy cells”? This is unclear and needs to be clarified.
• The TNT’s shown in the imaging don’t appear to be between cells and are not convincing. Rather than just pointing to a tube and suggesting it is a nanotube there needs to be some more clear and convincing imaging and co-labelling done.

Figure 3/4
• A key conclusion is that precursor stalling and expression of TauP301L have a common mechanism, but there is no evidence to support that the DHFR fusion has reached mitochondria, has stalled at TOM and is blocking import. There is absolutely no proof of concept in this regard and it is hard to rationalise how any of the data associated with the DHFR system is genuine without this proof of concept information.

First revision

Author response to reviewers' comments

Dear Prof. Schiavo,

Thank you and the reviewers for taking the time to evaluate our paper and provide constructive feedback. We have now revised the manuscript in line with the reviewers’ suggestions to address their concerns. As requested, we conducted an additional experiment (which worked!) and provided a few more controls where needed. Crucially, the new experiment reinforces our model and we now feel much more confident in our conclusions; so a particular thanks to reviewer 1 for this suggestion.

The new results are set alongside additional changes guided by the reviewers, as well as unsolicited changes for further improvements throughout the text.

We are confident that the new version is now much better, by virtue of your recommendations, and ready for publication in *JOCS*. We include below a point-by-point response to the reviewers’ comments to go with the revised manuscript. In the MS and supplementary information files, the new sections, incorporating changes in response to the reviewers, are highlighted.

We very much look forward to your views on our revision.

Sincerely,

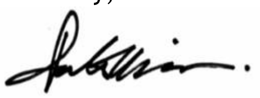

Ian Collinson, also on behalf of Prof Jeremy Henley.

© 2023. Published by The Company of Biologists under the terms of the Creative Commons Attribution License (https://creativecommons.org/licenses/by/4.0/).

Reviewer 1 Advance Summary and Potential Significance to Field:

Needs et al. have investigated the impact of pathogenic Tau mutant (TauP301L) on mitochondrial function and neuronal complexity. This is an important question and of broad relevance. The authors propose that TauP301L specifically interacts with the mitochondrial import machinery, which mildly disturbs mitochondrial morphology but does not impair mitochondrial import efficiency or OXPHOS. They observe tunnelling nanotubes (TNTs) in HeLa cells expressing TauP301L and suggest that organelle exchange via TNTs is required to maintain mitochondrial quality and import efficiency. Primary hippocampal neurons expressing TauP301L have fewer processes and dendritic synapses, which the authors attribute to defective mitochondrial import since they observe similar effects in neurons with artificially blocked mitochondrial import.

Previous studies have described various impacts of Tau on mitochondrial function and it is indeed interesting to consider the deleterious effect that mutant Tau may have on mitochondrial import efficiency. However, in my opinion the authors do not currently provide evidence to support their key claims that mutant Tau can disturb import or that this is the reason for reduced neuronal complexity.

Reviewer 1 Comments for the Author:

Main points

In Fig. 1, the authors blot TauP301L in mitochondrial fractions and identify an interaction with TOM40 by IP. There is no effect on mitochondrial import (S2) or oxygen consumption (S3) in TauP301L expressing cells. I would argue that any interaction between TauP301L and TOM40 does not necessarily mean that pathogenic Tau perturbs mitochondrial import, which is the central message of the manuscript. In Fig. 2, the authors present evidence of TNT formation and state that these can likely rescue import deficiencies in TauP301L expressing cells. A key experiment would be to perform import assays in TauP301L expressing cells that are unable to form TNTs (e.g as performed in the recent Needs et al., 2022 with the DHFR + MTX assay and nocodazole). This is required to test whether TauP301L can block import.

Excellent idea! We thank the reviewer for this suggestion. We carried out NanoLuc analysis, as recommended by the reviewer, to investigate the effect of Tau^{P301L} on import when the cell's ability to form TNTs is diminished. In keeping with our hypothesis, we saw that mitochondrial import was indeed reduced in cells expressing Tau^{P301L} in the presence of Nocodazole. This indicates that the association of Tau^{P301L} with TOM40 is affecting import function, as we observed for the trapping system in our recent preprint. Words to this effect have been added to the text along with this new data (Fig 2E, F).

Unfortunately, similar issues arise in the primary neuron work. The effects of TauP301L on neuronal complexity are entirely correlative with the artificial import blocker experiments, which the authors assume to be evidence of similar consequence to perturbed mitochondrial import, which is not shown in these cells. I appreciate it would be difficult to abolish TauP301L interaction with TOM40 in neurons, without knowing what drives this interaction, but this would help argue that the interaction between TauP301L and TOM40 is relevant to neuronal complexity.

We tend to agree it would be very challenging to eliminate the Tau^{P301L}-Tom40 interaction in neurons, but we do show that the interaction is reduced or abolished in HeLa cells.

Our observations are indeed correlative; the interaction Tau^{P301L} with TOM40, and its reduction (native Tau), are correlated with neuronal morphological changes. It is compelling that the perturbation of TOM40 in another way with a targeted trap has the same effect. However, we concede that the point has not been proven, and we now clearly say so in the text.

© 2023. Published by The Company of Biologists under the terms of the Creative Commons Attribution License (https://creativecommons.org/licenses/by/4.0/).

Other points

•The interaction between TauP301L and TOM40 is also not entirely convincing as presented. Is the IP in Fig1D on a single membrane (as shown for controls in Fig S1) and what is the % input?

The IP is on the same membrane, but the exposure is different since the abundance was very different in the input compared to the pulldown (as expected, very little of the total mitochondrial TOM40 is pulled down). We have now clarified this in the figure legend. Furthermore, to help clarify the result and convince the reader, we have quantified the Tau-TOM40 association, which shows a statistically significant association of Tau^{P301L} with TOM40, compared to Tau^{WT} or the GFP control. All the IP data has been added to the same figure, to make it clearer to the reviewers and readers the point we are trying to make - that TOM40, but no other tested mitochondrial proteins, is pulled down with Myc-Tau^{P301L}, but not with Myc-GFP or Myc-Tau^{WT}. The data is now displayed in Fig. 1D with quantification in supplementary Fig. S1B.

What is the evidence that the TOM40 interaction is specific as the authors claim, apart from not interacting with TOM20 and TIM23 (S1)?

Most likely, Tau will be interacting with many proteins. All we wish to say is that the interaction of Tau^{P301L} with TOM40 is stronger than with the native version (Fig. 1D, S1B) and that the interaction is specific to TOM40 (compared to TOM20 and TIM23). Moreover, a non-specific interaction would unlikely be affected by a single amino acid change of Tau (P301L). Note also that the interaction is specific to Tau as the Myc-GFP control is indeed negative.
To further support the specificity of the interaction, we performed an additional (non-import related) mitochondrial control - VDAC, which again was not pulled down with Myc-GFP, Myc-Tau^{WT}, or Myc-Tau^{P301L} containing samples (Fig. 1D).

Furthermore, can the authors also comment on the minimal colocalization between TauP301L and mitochondria in Fig 2C - confocal colocalization of TauP301L and the mitochondrial network would support the fractionation data and may give further clues to the inclusion of TauP301L decorated mitochondria in TNTs.

We agree that there is very minimal colocalization between Tau^{P301L} and the mitochondria observed by confocal microscopy, but this is not unexpected. Tau has major roles in microtubule stabilisation, and we only observed a very small proportion of Tau^{P301L} associating with mitochondria in our biochemical analyses (Fig. 1A). Thus, while difficult to observe with standard confocal microscopy, we contend that we identify a small but clear subpopulation of Tau^{P301L} that localises to mitochondria and has profound effects on import. The text has been modified to better explain this point. We also expand this imaging to confirm that the distribution of both variants of Tau is mostly non-mitochondrial (as expected).

•The same cell with mito-dsRed appears to have been used for images in Fig 2C and Fig. 1F. The authors should mention this.

We thank the reviewer for pointing this out and have now mentioned this in the text.

•Are stats performed on “biological replicates” (as I think should be) or individual cells e.g in Fig 1G legend there are two definitions of the N number?

Stats are always performed on 3-5 biological replicates (i.e., independent experiments). However, to be clear on how many objects were analysed per biological replicate, we notated the n-numbers as N = biological replicate (independent cell culture (i.e., different split in the case of HeLas, different animal/dissection in the case of neurons), n= analysed object (i.e., cell or mitochondria). This has now been clarified in the relevant figure legends as well as in the materials and methods section.

© 2023. Published by The Company of Biologists under the terms of the Creative Commons Attribution License (https://creativecommons.org/licenses/by/4.0/).

Reviewer 2 Advance Summary and Potential Significance to Field:

The study by Needs and colleagues explores mitochondrial protein-import dysfunction as a cause of neurodegeneration by exploiting a Tau model of neurodegeneration. The authors suggest:

•a Tau mutant - TauP301L binds to the protein-channel of the outer-membrane TOM40
•the interaction between TauP301L and Tom40 affects mitochondrial morphology, but does NOT impact mitochondrial import, or respiratory function

•TauP301L induces the formation of tunneling nanotubes (TNTs)

•primary neuronal cultures show morphological changes characteristic of neurodegenerative disease in the presence of TauP301L

•changes to neuronal structure are described when mitochondrial import is blocked artificially.

Reviewer 2 Comments for the Author:

The authors conclude perturbations in mitochondrial protein import, specifically blockage of the central entry gate TOM complex may be an important factor in the onset of neurodegenerative diseases. This aligns with previous suggestions in the literature and is an exciting avenue of research, but unfortunately key experiments in this manuscript do not actually support this conclusion and have been over interpreted to align with this proposed model. The paper presents an exciting concept worthy of consideration, but would require significant revision to be at a standard for JOCES.

The paper is now significantly revised, including additional experiments (see the responses to reviewer 1 and specific points below). The experiment incorporating Nocodazole (Fig 2E, F) was particularly useful. By diminishing the TNTs (and import rescue) we show that Tau has an impact on import (more so with Tau^{P301L} compared to Tau^{WT}). This observation supports our model and the conclusion of the paper (outlined in the response above).

General items

[1] The title does not reflect the findings of the study. The research does not demonstrate effectively an import defect in the presence of mutant Tau. Indeed the authors show and interaction of mutant Tau with Tom40 and that import into the matrix (Fig 1E) is unaffected, themselves concluding that there is no import defect. The use of the term perturbation of protein import in the title and general conclusions of this paper is not appropriate and should be modified.

We have now carried out NanoLuc analysis to investigate the effect of the Tau amino acidsubstitution P301L on import when cells are unable to form TNTs (Fig 2E, F). In keeping with our hypothesis, we saw that mitochondrial import was reduced in cells expressing Tau^{P301L} in the presence of Nocodazole. This indicates that Tau^{P301L} association with TOM40 does indeed bring about the ‘perturbation’ of import, correlated with a loss of neuronal complexity. This is quite compelling, given that a trapped precursor has the same effect on cultured neurons.

Nevertheless, we acknowledge that the link between import blocking and neurodegeneration is not yet proven and have added a note of caution to the text.

[2] The Materials and Methods are lacking clarity and information to guide the reader. The results section needs to be more explicit in some sections. For example Fig 1A, are these constructs introduced into HeLa cells using transient transfection or the lentiviral system described in the methods. The methods are lacking specific detail making it hard for the reader to comprehend what has been done where. As one example the lentiviral infection - which genes were clones into these vectors, which cells lines was this system used on? Why is there a section describing purification of GST proteins as it is not clear from the results where there was any GST proteins

© 2023. Published by The Company of Biologists under the terms of the Creative Commons Attribution License (https://creativecommons.org/licenses/by/4.0/).

applied. What was the GST Dark peptide used for? There is no information on the DHFR blocking experiments and this is vital to understand what has been undertaken to come to the conclusion that there is blockage in the neurons. This methods seems to have been scrambled together and lack general QC.

We thank the reviewer and have acted on their advice. The Materials and Methods and Results sections have now been edited to be clearer on how exactly experiments were done. Specifically, we have clarified in the text where transfection or infection was used. We have already stated how the genes for lentiviral infection were cloned, see the end of the generation of constructs section: *('Specifically, human Tau 4R0N isoform WT or P301L coding sequences were amplified from pSinRep5-EGFP-Tau expressing plasmids (a gift from Prof Neil Marrion, University of Bristol) by PCR and cloned with an N-terminal Myc-tag into the SpeI and BamHI sites of the lentiviral plasmid pXLG3-PX-WPRE.')*. We have clarified within the text what the proteins mentioned (including GST-dark) were used for, and have made significant reference to a Methods paper (Needs et al., 2023) describing this technique in detail. We have now added a section describing how DHFR-MTX trapping is achieved and referenced previous studies that have used this trapping method.

[3] In general I am not comfortable with significant reference to a concurrent pre-print of research that aligns with this paper - that has yet to be peer reviewed. This is something for editorial consideration.

The paper related to in-cell mitochondrial import monitoring is now published (Needs et al., 2023). The referenced pre-print relating to TNTs is currently under peer review at *Nat. Comms.* (98 days!) and is readily accessible on BioRxiv (Needs et al., 2022). We note that *JOCS* policies support and encourage the citation of relevant pre-prints in their publications.

Comments pertaining to Data:
Figure 1

•method used for determining protein localisation % to the mitochondria is not clear. 1A should show a Total sample and how the cytosol and mitochondria compare in this regard.

We thank the reviewer, and this has now been reanalysed to show the proportion of the protein in the mitochondrial and cytosolic fractions (Fig. 1B), which we agree makes it clearer. The text has been edited to clarify exactly how this has been done.

•Figure 1A - Interesting that P301L expressing is impacting the levels of Tom20 and Tim23 but the protein is interacting with Tom40. Do and how do the levels of the TOM and TIM23 complexes change by BN-PAGE.

We thank the reviewer for their insightful comment and agree that this would be a good experiment for a follow-up study; this has now been mentioned in the discussion section. However, this analysis is beyond the scope of this paper and is not absolutely necessary for the main story.

•Figure 1D is not properly controlled. Supp Figure S1 should be shown in the main Figure. These blots all need loading controls - other mitochondrial proteins to show that something like Tom40 is not coming down non-specifically. IP coupled with mass spectrometry would be a more valuable and non-biased approach to deciphering how the interaction of wild-type and mutant Tau differ.

As requested, we have now added all the Western blots to Fig. 1D.

In terms of specificity, the Western blots show that whilst TOM40 is pulled down with Tau^{P301L}, neither TOM20 nor TIM23 are pulled down, highlighting the specificity of the TOM40-Tau^{P301L} interaction. We have now also probed for VDAC, a classical mitochondrial loading control, which is not pulled down with Tau^{P301L}.

© 2023. Published by The Company of Biologists under the terms of the Creative Commons Attribution License (https://creativecommons.org/licenses/by/4.0/).

We agree that IP coupled with mass spectrometry would be a useful, unbiased approach to obtain similar results, and this would be a good experimental approach for follow-up studies. Remarks on this have now been added to the discussion.

•The panel for Tom20 (input) in Fig S1 does not align with the conclusion made in Figure 1A. In 1A levels of Tom20 are reduced in the presence of mutant Tau, while Supp S1 shows the opposite. How can the authors rationalise the conclusion made in 1A.

We apologise for this oversight, on looking more closely at this blot it appears not to be a representative blot, as there appears to be a bubble on the membrane. This has now been replaced with a more representative blot, that is in line with the conclusions drawn, and the corresponding biological replicate has been removed from the quantification. This amendment did not affect the original interpretation, that the levels of TOM20 are significantly reduced along with Tau^{P301L} (Fig. 1C).

•it is concluded that there is an import defect (in reference to abundance of mitochondrial import subunits), but direct evidence of an import defect is lacking. Import rates may be unaffected by these small changes in subunit abundances and only one import pathway is tested with the assay in 1E (matrix import) - what about other pathways into mitochondria? If import defect is the mechanism at play this needs to be interrogated and shown with a more reliable method. For example how does the mitochondrial proteome change in the presence and absence of mutant Tau?

As mentioned for general comment [1], we have now carried out MitoLuc analysis to investigate the effect of Tau^{P301L} on import when cells are unable to form TNTs. In keeping with our hypothesis, we saw that mitochondrial import was reduced in cells expressing Tau^{P301L} in the presence of Nocodazole. This points to Tau^{P301L} association with TOM40 leading to mitochondrial protein import dysfunction, as we observed for the trapping system in our recent preprint. Words to this effect have been added to the text along with this data (Fig 2E, F).

These data show a direct effect of Tau^{P301L} on import *via* the presequence pathway. We agree that it would be interesting to observe if/how import *via* the other pathways is altered. However, we do not currently have the tools to do so. We have commented on this in the discussion. Likewise, looking at mitochondrial proteome changes would be a very interesting set of experiments for a follow-up study to this paper; this point has been noted in the discussion section.

•There is no positive control for the NanoLuc assay. Can the readers be reassured that the assay is working?

The Su9-EGFP-pep86 is one of our standard precursor test samples for the MitoLuc assay, fully characterized in the now published (Needs et al., 2023). We know that it works so it has become a positive control. Furthermore, the presence of GFP will not affect import. Thus, Su9-EGFP-pep86 import in the presence of GFP alone is in effect the positive control. It is the best option to use in this case, to control for the presence of GFP, and thereby measure only the effects of the Tau variants. Additionally, we have used this as a consistent control throughout the study. We have now clarified this point in the text.

Figure 2

It is not clear if the whole population off cells shown here are expressing the GFP-TAU wild-type or mutant. Are these cells transiently transfected or transduced with virus and selected. If the entire population is expressing the Tau variant what purpose would TNT's serve if all the cells are unhealthy? How could the mechanism of healthy/unhealthy mitochondrial exchange occur if there are no “healthy cells”? This is unclear and needs to be clarified.

© 2023. Published by The Company of Biologists under the terms of the Creative Commons Attribution License (https://creativecommons.org/licenses/by/4.0/).

We thank the reviewer for pointing this out; this has now been clarified and discussed in more detail in the text to ensure it is clear to the reader.

Expression is achieved by transfection, meaning there will be a subset of cells that express the Tau variant (cells with challenged mitochondria), and another subset not expressing the Tau variant (healthy cells with fully functional mitochondria).

We propose that TNTs form between healthy and challenged cells and transfer healthy mitochondria into cells with Tau accumulation, thereby providing a mechanism to rescue their mitochondrial import function. It is likely that the TNTs also transfer dysfunctional mitochondria away from the challenged cells, possibly for degradation, although we have not yet tested directionality in this system.

•The TNT's shown in the imaging don't appear to be between cells and are not convincing. Rather than just pointing to a tube and suggesting it is a nanotube there needs to be some more clear and convincing imaging and co-labelling done.

We accept the first images were not entirely compelling and have done further imaging experiments where cells were transfected with Myc-GFP, Myc-Tau^{WT}, or Myc-Tau^{P301L}. Cells were stained with wheat germin agglutin (WGA), which is a membrane marker commonly used to identify TNTs (Benard et al., 2015). We co-stained against Myc to highlight transfected and untransfected cells. The resulting images show more clearly that what we are highlighting in the other figures are indeed TNTs. The data is shown in supplementary Fig. S2A and text has been added to describe this.

Figure 3/4

•A key conclusion is that precursor stalling and expression of TauP301L have a common mechanism, but there is no evidence to support that the DHFR fusion has reached mitochondria, has stalled at TOM and is blocking import. There is absolutely no proof of concept in this regard and it is hard to rationalise how any of the data associated with the DHFR system is genuine without this proof of concept information.

As referenced in the text, the DHFR-MTX trapping system is a widely used and well-established import trapping system (Chacinska et al., 2003; Eilers and Schatz, 1986; Ford et al., 2022; Rassow et al., 1989). We have now added another comment about this to the text in the neuron section and note that in the neuronal images (Fig. 3D, 4C), in the presence of MTX, the Su9-EGFP-DHFR trapping substrate is mostly excluded from the mitochondria of the neurons, showing that the trapping is working.

Furthermore, we do show proof of concept experiments in our companion preprint/paper (Needs et al., 2022) and cite many previous reports (as above). Specifically, we show the exclusion of the trap substrate from the mitochondria of HeLaGAL cells (by confocal imaging, SIM, and Western blot), and a concurrent reduction in import (by MitoLuc import assays). We feel that replicating data that has been validated elsewhere, both by us and many others, is not an efficient or necessary use of resources.

References

Benard, M., Schapman, D., Lebon, A., Monterroso, B., Bellenger, M., Le Foll, F., Pasquier, J., Vaudry, H., Vaudry, D. and Galas, L. (2015). Structural and functional analysis of tunneling nanotubes (TnTs) using gCW STED and gconfocal approaches. *Biology of the Cell* **107**, 419-425.

Chacinska, A., Rehling, P., Guiard, B., Frazier, A. E., Schulze-Specking, A., Pfanner, N., Voos, W. and Meisinger, C. (2003). Mitochondrial translocation contact sites: separation of dynamic and stabilizing elements in formation of a TOM-TIM-preprotein supercomplex. *Embo Journal* **22**, 5370-5381.

© 2023. Published by The Company of Biologists under the terms of the Creative Commons Attribution License (https://creativecommons.org/licenses/by/4.0/).

Eilers, M. and Schatz, G. (1986). BINDING OF A SPECIFIC LIGAND INHIBITS IMPORT OF A PURIFIED PRECURSOR PROTEIN INTO MITOCHONDRIA. *Nature* **322**, 228-232.

Ford, H. C., Allen, W. J., Pereira, G. C., Liu, X., Dillingham, M. S. and Collinson, I. (2022). Towards a molecular mechanism underlying mitochondrial protein import through the TOM and TIM23 complexes. *Elife* **11**.

Needs, H. I., Lorriman, J. S., Pereira, G. C., Henley, J. M. and Collinson, I. (2023). The MitoLuc Assay System for Accurate Real-Time Monitoring of Mitochondrial Protein Import Within Mammalian Cells. *Journal of Molecular Biology* **435**, 168129.

Needs, H. I., Pereira, G. C., Glover, E., Witt, A., Hübner, W., Dodding, M. P., Henley, J. M. and Collinson, I. (2022). Intercellular Mitochondrial Transfer as a Rescue Mechanism in Response to Protein Import Failure. *bioRxiv*, 2022.11.30.518494.

Rassow, J., Guiard, B., Wienhues, U., Herzog, V., Hartl, F. U. and Neupert, W. (1989). TRANSLOCATION ARREST BY REVERSIBLE FOLDING OF A PRECURSOR PROTEIN IMPORTED INTO MITOCHONDRIA - A MEANS TO QUANTITATE TRANSLOCATION CONTACT SITES. *Journal of Cell Biology* **109**, 1421-1428.

Second decision letter

MS ID#: JOCES/2023/260993

MS TITLE: Perturbation of mitochondrial import by Tau affects organelle morphology and neuronal complexity

AUTHORS: Hope I Needs, Kevin Wilkinson, Jeremy Henley, and Ian Collinson
ARTICLE TYPE: Research Article

We have now reached a decision on the above manuscript.

To see the reviewers' reports and a copy of this decision letter, please go to: https://submit-jcs.biologists.org and click on the 'Manuscripts with Decisions' queue in the Author Area. (Corresponding author only has access to reviews.)

As you will see, the reviewers gave favourable reports but a reviewer raised some critical points that will require amendments to your manuscript. I hope that you will be able to carry these out because I would like to be able to accept your paper, depending on further comments from reviewers.

Please ensure that you clearly highlight all changes made in the revised manuscript. Please avoid using 'Tracked changes' in Word files as these are lost in PDF conversion.

I should be grateful if you would also provide a point-by-point response detailing how you have dealt with the points raised by the reviewers in the 'Response to Reviewers' box. Please attend to all of the reviewers' comments. If you do not agree with any of their criticisms or suggestions please explain clearly why this is so.

© 2023. Published by The Company of Biologists under the terms of the Creative Commons Attribution License (https://creativecommons.org/licenses/by/4.0/).

Reviewer 1

Advance summary and potential significance to field

The authors have addressed my comments in good faith and the MitoLuc experiment (Fig. 2E) does strengthen the argument that mutant Tau impacts import, which remains correlative with a Tom40 interaction. New additions to the text help reduce some over interpretation of the data.

Comments for the author

I would still encourage the authors to reword the title. The discussion now states that “It is important to note that the association of TauP301L with the import machinery was shown in HeLa cells and not in neurons, and as such the implications for neurons concerning import dysfunction are solely correlative.” To me, this seems at odds with the definitive title.
Although Fig 1D has been improved, my point regarding the IP data was not entirely clarified in the figure legend. The authors should state that they used different exposure times for the IP vs input, which will emphasise to the reader that it is a very minor fraction of TOM40. (Though I’m surprised the exposure times are different because there is no obvious change in background intensity between input and IP lanes). Ideally an indication of the % input loaded would also help the reader interpret the data.

Reviewer 2

Advance summary and potential significance to field

N/A

Comments for the author

The comments have been adequately addressed. The manuscript should be accepted for publication.

Second revision

Author response to reviewers' comments

Reviewer 1 Advance Summary and Potential Significance to Field:
The authors have addressed my comments in good faith and the MitoLuc experiment (Fig. 2E) does strengthen the argument that mutant Tau impacts import, which remains correlative with a Tom40 interaction. New additions to the text help reduce some over interpretation of the data.

Reviewer 1 Comments for the Author:
I would still encourage the authors to reword the title. The discussion now states that “It is important to note that the association of TauP301L with the import machinery was shown in HeLa cells and not in neurons, and as such the implications for neurons concerning import dysfunction are solely correlative.” To me, this seems at odds with the definitive title.

We have changed the title of our manuscript: *Aggregation prone Tau impairs mitochondrial import, which affects organelle morphology and neuronal complexity*. The claims that Tau impairs import, and import impairment affects mitochondrial morphology and neuronal complexity have been demonstrated in the MS.

Although Fig 1D has been improved, my point regarding the IP data was not entirely clarified in the figure legend. The authors should state that they used different exposure times for the IP vs input, which will emphasise to the reader that it is a very minor fraction of TOM40. (Though I’m surprised

© 2023. Published by The Company of Biologists under the terms of the Creative Commons Attribution License (https://creativecommons.org/licenses/by/4.0/).

the exposure times are different because there is no obvious change in background intensity between input and IP lanes). Ideally an indication of the % input loaded would also help the reader interpret the data.

The input represents 25% of the lysate used in the pulldown. We have now clarified this in the figure legend for Fig. 1D and stated that different exposures were used for the input and the pulldown. Note that both exposures are also shown in the 'blot transparency' figure, Fig. S4.

Reviewer 2 Advance Summary and Potential Significance to Field:
N/A

Reviewer 2 Comments for the Author:
The comments have been adequately addressed. The manuscript should be accepted for publication.

Third decision letter

MS ID#: JOCES/2023/260993

MS TITLE: Aggregation prone Tau impairs mitochondrial import, which affects organelle morphology and neuronal complexity

AUTHORS: Hope I Needs, Kevin Wilkinson, Jeremy Henley, and Ian Collinson
ARTICLE TYPE: Research Article

I am happy to tell you that your manuscript has been accepted for publication in Journal of Cell Science, pending standard ethics checks.

© 2023. Published by The Company of Biologists under the terms of the Creative Commons Attribution License (https://creativecommons.org/licenses/by/4.0/).
